# Adhesive bond durability and corrosion resistance of AA6060-T6 flash-PEO treated in Mo-Si solutions

**DOI:** 10.1038/s41529-026-00841-5

**Published:** 2026-07-15

**Authors:** Dominic Shore, Junia C. Avelar-Batista Wilson, Nicolas Laugel, Allan Matthews, Aleksey Yerokhin

**Affiliations:** 1 Department of Materials, University of Manchester, Manchester, UK; 2 Henry Royce Institute, University of Manchester, Manchester, UK; 3 BCW Treatments Ltd, Burnley, UK

**Keywords:** Chemistry, Engineering, Materials science

## Abstract

Aluminium adhesive bonding is a key enabler in lightweighting engineering structures, directly influencing global energy consumption and carbon emissions. Plasma electrolytic oxidation (PEO) has recently emerged as an environmentally friendly alternative to industry-standard sulphuric acid anodising (SAA) surface preparation prior to bonding. However, little is known about the longer-term durability of adhesively bonded PEO-treated structures. Here, AA6060-T6 Al alloy samples were subjected to unconventionally brief 70-s flash-PEO treatments using high-frequency 2.5 kHz unipolar and bipolar pulsed polarisation in aqueous alkaline electrolytes containing Na_2_SiO_3_ and optionally Na_2_MoO_4_. Molybdate addition and bipolar polarisation improved uniformity and corrosion resistance of thin, ~1 μm, oxide layers produced. All studied flash-PEO coatings performed well in lap shear tests, exhibiting adhesive strength comparable to that of sealed SAA films and demonstrated equivalent or enhanced bond durability when tested after 1008-h Sea Water Acetic Acid Test exposure, indicating satisfactory resistance to moisture and corrosion with preferential adhesive fracture. Comparable to SAA, the long-term bonding performance of flash-PEO treatments highlights their potential as a sustainable surface preparation route for aluminium adhesive bonding.

## Introduction

Driven by the improvements to efficiency and performance that can be achieved through lighter weight designs, the application of aluminium alloys for automotive components has grown and is expected to continue to rise rapidly in the future with the emergence of technological trends such as electric vehicles^1,2^. Due to the difficulties in joining aluminium components via traditional joining techniques such as welding, many structural aluminium components are joined via adhesive bonding with structural epoxy adhesives. Structural automotive components are expected to safely endure the duration of their vehicle lifetime without exhibiting a depreciation in structural integrity. Whilst adhesive bonding provides excellent strength, stiffness, stress distribution and manufacturability^3–5^, long-term durability is a key concern when assessing the long-term performance of bonded aluminium structures. It is of paramount importance that adhered components exhibit bond surfaces with suitable physical and chemical properties promoting sufficient wettability, adsorption and mechanical interlocking whilst being sufficiently resistant to corrosion, a major concern of reduction in adhesive bond strength.

To promote long-term durability, bonded structural aluminium components are typically subjected to surface treatments. In the case of structural automotive components, a sulphuric acid anodising (SAA) treatment with a hydrothermal sealing post-treatment may typically be applied to promote enhanced corrosion resistance and, as a result, enhanced bond durability^6–8^. The anodic alumina coatings provide excellent corrosion resistance, and their metal oxide/hydroxide surface composition is suitable for adsorption adhesion^9,10^. Whilst SAA coatings exhibit good performance, the anodising technology has several drawbacks, including requiring a number of pre-treatment and post-treatment steps to achieve the requisite coating and the use of harsh, environmentally harmful chemicals at several stages of the process^11^.

Plasma Electrolytic Oxidation (PEO) is an electrolytic process born out of conventional anodising where anodic potentials surpass the dielectric breakdown voltage of the growing oxide, resulting in plasma discharge events on the surface, which contribute to the growth and modification of the growing oxide coating. Crystalline oxide layers with thicknesses of up to hundreds of microns can be produced on valve metals, including aluminium, providing excellent wear and corrosion resistance^12–15^. PEO has the advantage of utilising environmentally benign weakly alkaline electrolytes, and the robust sparking renders the process less sensitive to inconsistencies in surface composition, meaning that pre-treatment steps are not necessarily required. The physical and chemical properties of PEO coatings can be optimised through tailoring of the electrical polarisation signal and electrolyte composition. The ability to deposit electrolyte species into the coating allows for further functionalisation during processing^16^. There are still many unknowns in the collective understanding of the mechanistic principles underlying the PEO process, which have hindered its widespread industrial implementation.

In the study presented, unconventionally thin (~1 μm), ‘flash’ PEO coatings produced via short treatment times were assessed for their suitability for the surface treatment of AA6060-T6 series aluminium alloy for adhesive bonding applications. Flash-PEO coatings have been researched on alloys of aluminium and magnesium for the promotion of enhanced corrosion resistance and paint adhesion^17–20^. It is envisaged that utilisation of short treatment times could reduce the difference in energy consumption compared to anodising treatments, whilst enabling streamlining of the whole process, yet potentially still providing suitable adhesion and corrosion properties. However, performing short PEO treatments of Al provides a major challenge in terms of achieving desirable process consistency with uniform thickness and homogeneous surface finish of such thin oxide layers grown under the influence of localised microdischarge events. Unfortunately, most studies bypass this issue by reporting on much thicker (~10s μm) PEO coatings on Al grown for 10s of minutes rather than seconds.

A limited amount of previous work has been undertaken investigating plasma electrolytic treatments for adhesive bonding applications. We previously assessed the adhesive bond strength obtained for dense ceramic coatings in the range of 15–25 microns-thick produced via potentiostatic PEO processing of aluminium^11^. It was observed that thicker coatings failed at lower loading, underscoring the importance of research into unconventionally thin PEO coatings. The adhesive bond strength of PEO-treated joints has been studied for titanium^21,22^ and also for magnesium alloys^23,24^ where PEO was observed to significantly increase bond strength. However, these studies have been limited to assessing joint strength in the ‘as-new’ condition. To the best of our knowledge, there are no published studies on the application of flash-PEO treatments for adhesive bonding of Al structures, nor on the evaluation of the long-term durability of bonded PEO-treated surfaces in general. Bridging these knowledge gaps is essential for assessing the applicability of such technology for real-world applications where the long-term integrity of joints is often the main limiting factor.

In this study, flash-PEO type coatings were produced under unipolar and bipolar galvanostatic processes in two aqueous alkaline electrolytes, one consisting of sodium silicate (Na_2_SiO_3_) and the other consisting of Na_2_SiO_3_ and sodium molybdate (Na_2_MoO_4_). With a view to commercial scalability, the treatments were carried out at modest applied current densities compared to typical examples of PEO treatments of Al alloys^25^. This work presents one of the first investigations into the application of pulsed reversed (bipolar) polarisation during flash-PEO (70 s) processing of an aluminium alloy. The motivation behind the utilisation of sodium molybdate (a well-known corrosion inhibitor for metals, including aluminium^26,27^) as an electrolyte addition for PEO processing was to assess the implications of its addition on the corrosion resistance of produced coatings and its effect on the process phenomenology and the enablement of uniform processing in short-duration treatments. Whilst compounds of tungsten (a similar group-VI transition metal) have been widely investigated as an addition to silicate electrolytes for corrosion-resistant PEO coatings on aluminium^28,29^, studies on molybdate-containing electrolytes for corrosion-resistant PEO coatings on Al are much scarcer. It has been shown that silicate-molybdate–zirconia electrolytes can improve polarisation resistance of pulsed unipolar PEO coatings due to morphological changes^30^, whilst those produced in tartrate-borax-phosphate-molybdate, and molybdate-titanate electrolytes have also been demonstrated to exhibit good corrosion resistance^31^. However, both studies assessed only a short-term (~1 h) corrosion performance of relatively thick oxide layers, leaving the question of molybdate efficiency in flash-PEO technology for thin coatings on Al wide open.

To assess the suitability of thin PEO coatings for adhesive bonding applications, flash-PEO treated Al-alloy samples were characterised via SEM, EDX, XRD, XPS, profilometry and contact angle measurement that enabled evaluation of the physical and chemical properties of surface oxide layers and specifically the topography and surface chemistry. To ascertain a better understanding of the corrosion resistance afforded by the treatments, an electrochemical investigation was performed on treated samples. Open circuit potential (OCP) and electrochemical impedance spectroscopy (EIS) measurements were recorded and compared for the different treatments. The adhesive bond strength and durability were assessed through lap shear testing of bonded samples prior and after accelerated ageing of the joints.

AA6060-T6 samples treated via a conventional sealed sulphuric acid anodising process were investigated alongside the PEO treatments studied in this work. The industry standard SAA-treated samples would act as a benchmark, offering a useful comparison for the performance of PEO treatments. Furthermore, there has been little in the way of published work on the performance of industrial SAA-treated AA6060-T6 with respect to corrosion properties, making the electrochemical evaluation of these samples of wider interest.

## Results

### Investigation into the edge effect

One of the commonly occurring detrimental phenomena associated with electrolytic surface treatments is uneven coating formation due to the so-called ‘edge effect’, which is observed in conventional electrochemical processes such as electroplating and is usually associated with increased current density at the corners and regions closer to the counter electrodes, leading to higher local coating deposition rates^32,33^. The edge effect is also prevalent in PEO treatments and is complicated further by the application of dynamic polarisation signal and associated non-Faradaic processes. The effect on aluminium alloys is exacerbated due to the high relative permittivity (*ε*_r_) of Al_2_O_3_^34^ compared to the electrolyte^35^. The occurrence of non-uniform sparking in the first minutes of PEO treatment under bipolar conditions is typical and likely to be one of the chief reasons limiting previous research on the application of bipolar regimes for short-duration surface treatments.

**Fig. 1:**
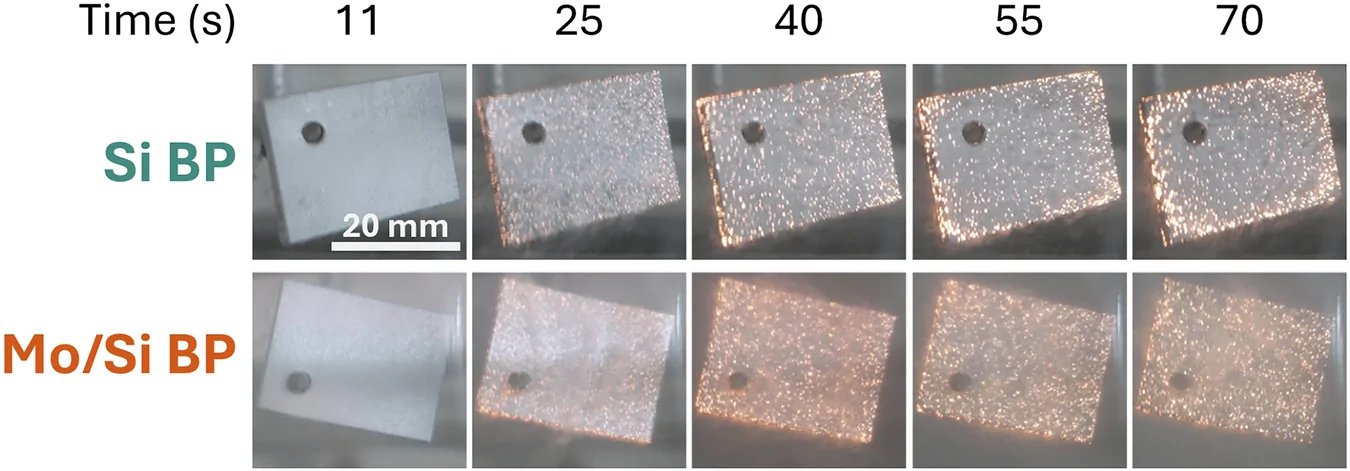
Flash-PEO discharges. Evolution of discharge appearance during bipolar flash-PEO treatments of AA6060-T6 in Si and Mo/Si electrolytes (sample size 25 mm × 20 mm).

**Fig. 2:**
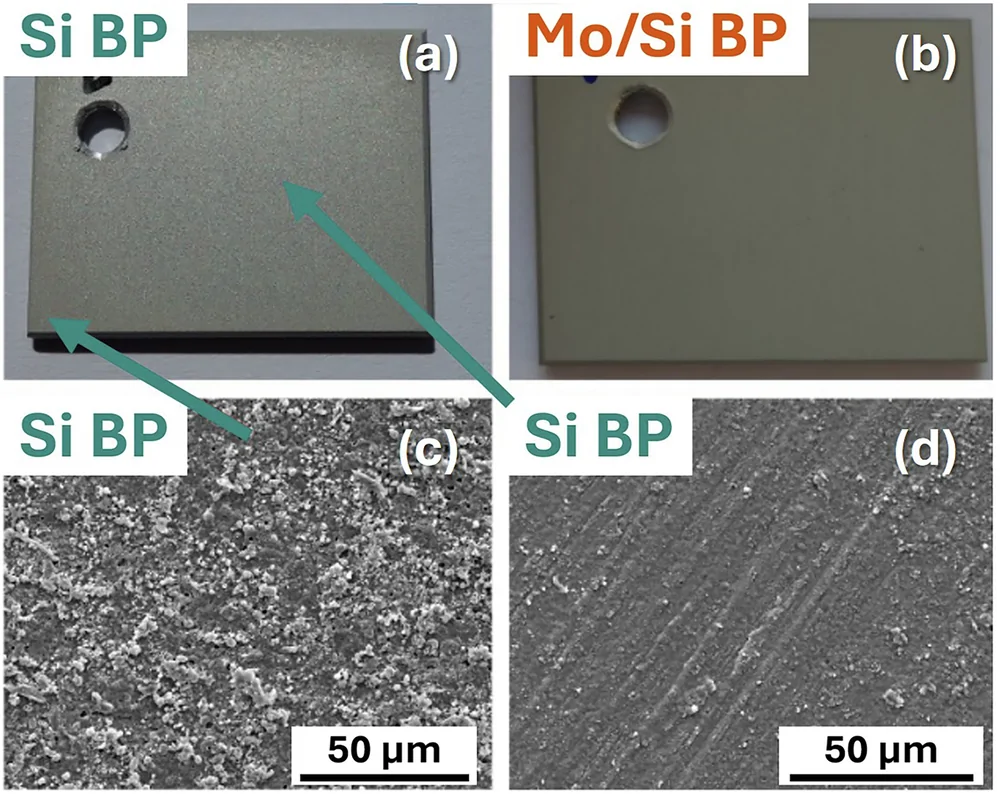
Macroscopic and microscopic surface appearance of flash-PEO-treated samples. **a** Edge effect on a non-uniform Si BP sample and **b** uniform Mo/Si BP sample (both samples are 25 mm × 20 mm in size). SEM micrographs of outer edge (**c**) and inner regions (**d**) of the non-uniform Si BP coating.

Voltage transients in the four studied flash-PEO conditions are provided in Supplementary Materials, Fig. S1, accompanied by a detailed discussion. For the two bipolar treatments undertaken in this work, different sparking behaviour was observed in the two studied electrolytes (Fig. 1). In the case of the Si electrolyte, the first sparking was observed ~12–13 s into the process. In the first 25 s, numerous small discharges initiated at the edge of the sample and very quickly propagated towards the centre of the flat surface. At 40 s, fewer but larger discharges or cascades of discharges emerged at the edges, whilst smaller, sparsely populated sparks were still seen in the central region of the flat surface. By the end of the treatment, the larger sparks were also seen gradually moving towards the centre, albeit most of them still clustered at the edges. In the case of the Mo/Si electrolyte, the process was initiated in a similar manner, with sparking occurring 12–13 s in the form of numerous light microdischarges. As the treatment progressed, the intensity of the sparks increased; however, a high density of uniformly distributed sparks was maintained across the surface. Typical surface appearance of samples subjected to the two bipolar PEO treatments is displayed in Fig. 2. The Mo/Si BP sample exhibited a homogeneous surface, whereas the Si BP sample showed two distinct surface morphologies where the two different sparking modes prevailed. The surface morphology observed at each of these regions under SEM is depicted in the bottom row. At the edge, a nodular surface morphology typical of aluminium PEO treated in silicate electrolytes is observed, whereas in the middle region, a smoother surface indicative of the lack of sparking can be seen.

**Fig. 3:**
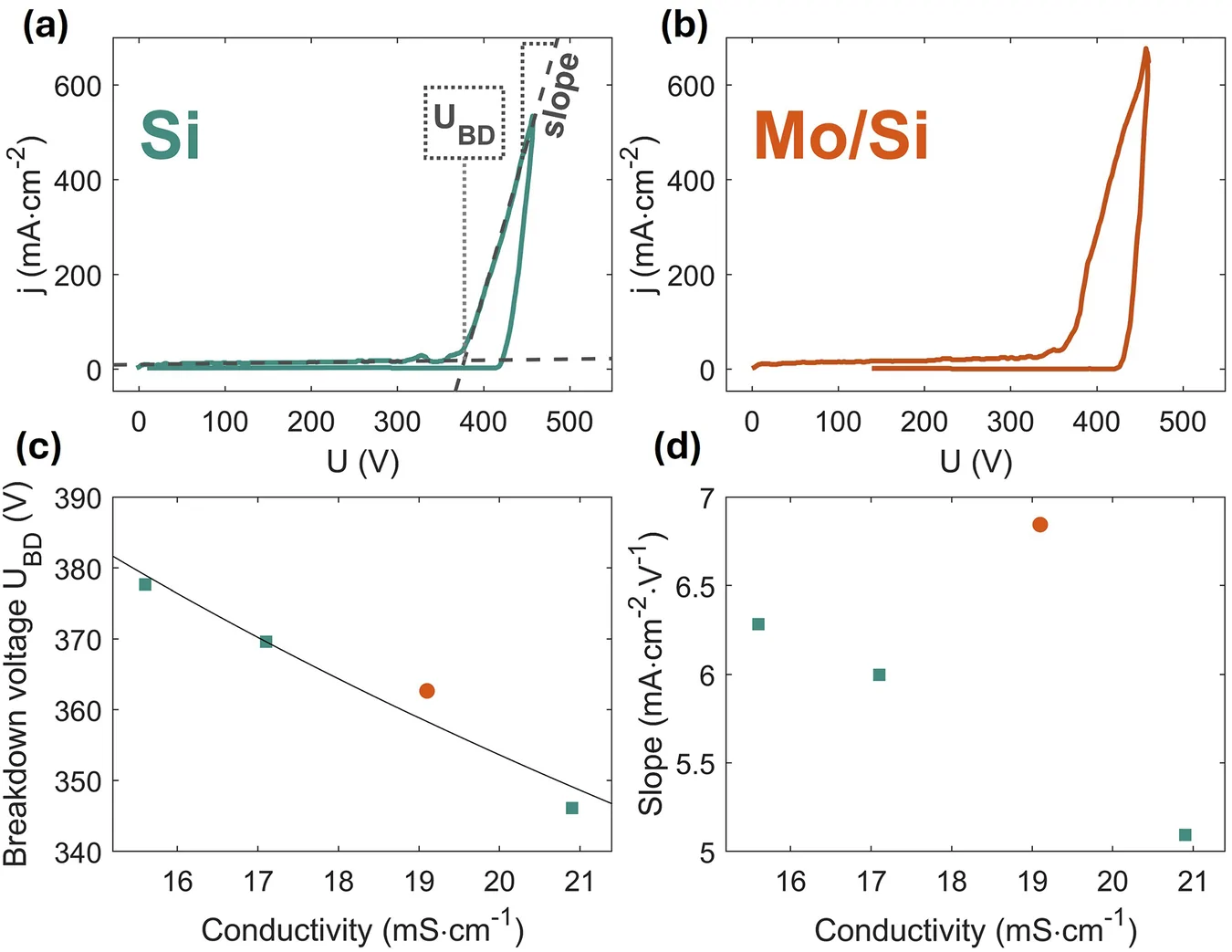
Electrochemical evaluation of the studied PEO processes. Cyclic voltammograms for AA6060-T6 in electrolytes **a** Si (0.05 M KOH and 0.118 M Na_2_SiO_3_) and **b** Mo/Si (Si with further addition of 0.025 M Na_2_MoO_4_). Characteristic breakdown voltage (**c**) and post-breakdown slope (**d**) derived from voltammograms taken in electrolytes Si at concentrations 0.118, 0.143 and 0.223 M Na_2_SiO_3_ (squares) and Mo/Si at concentrations 0.118 M/0.025 M (circles), respectively. All solutions pH = 12.4.

To elucidate the effect of electrolyte ionic conductivity on the edge effects at the earlier stages of PEO treatments, electrolytes based on 0.05 M KOH with additions of 0.143 and 0.223 M of Na_2_SiO_3_ that exhibited a higher ionic conductivity than the Mo/Si electrolyte were additionally utilised. The Al alloy samples treated under identical bipolar PEO processing conditions were found to still exhibit similar uneven sparking behaviour as the main Si electrolyte, albeit the effects were marginally less exaggerated with increasing conductivity. Anodic cyclic voltammograms (Fig. 3) show a decrease in breakdown voltage *U*_BD_ consistent with the Ikonopisov model^36^, following a logarithmic relationship with electrolyte resistivity (bottom left panel, black line) and remaining largely independent of electrolyte composition.

The voltammograms’ post-breakdown slopes vary with electrolyte composition due to multiple antagonistic factors. On the one hand, higher electrolyte conductivity is expected to steepen the slope by reducing electrolyte resistance *R*s. On the other hand, higher silicate concentration will lead to a larger silica fraction in the coating, which not only contributes to coating build-up without contributing to current, but also tends to block subsequent discharge pathways due to silica’s higher dielectric strength. Both can be expected to flatten the slope as silicate concentration and conductivity increase. Si electrolyte results show a decreasing trend and suggest that these differences in coating composition and thickness more than compensate for the lower *R*s. A parallel can be made with the Wagner number^37^ and throwing power^38^ concepts from traditional surface engineering electrochemistry, both correlating positively with electrolyte conductivity and negatively with the ∂*j*/∂*U* slope. In this light, our observations suggest that higher silicate concentrations will lead to a narrower current-density distribution over the active area, reducing edge effects and leading to more uniform coatings.

In the Mo/Si electrolyte, an active inhibitor is introduced in molybdates, promoting alumina formation and enhancing electrochemical activity and current flow. Additionally, different Mo-containing species may adsorb onto the coating, as evidenced by XPS (see Fig. 9 and discussion below), and act as redox-activating sites. This explains the steeper post-breakdown slope in Mo/Si compared to Si electrolytes and aligns with the more even discharge distribution observed in Fig. 1.

The mechanism by which the addition of Na_2_MoO_4_ improves sparking uniformity is not completely clear at this stage. It could be speculated that Mo incorporation into the coating may increase oxide permittivity^39–41^ which would alleviate the severity of the edge effect. It could conversely be speculated that Na_2_MoO_4_, which is a known corrosion inhibitor for Al, alters sparking behaviour by shifting the balance of anodic reactions at the metal–oxide–electrolyte junction towards oxide formation processes. In either case, the addition of molybdate to Si-based electrolytes could be useful in facilitating uniform processing under short durations, and it may prove more useful when scaling such processes up, especially when more complex substrate geometries will further complicate the primary electric field distribution in the electrolyser.

### Morphology and composition of PEO coatings

**Fig. 4:**
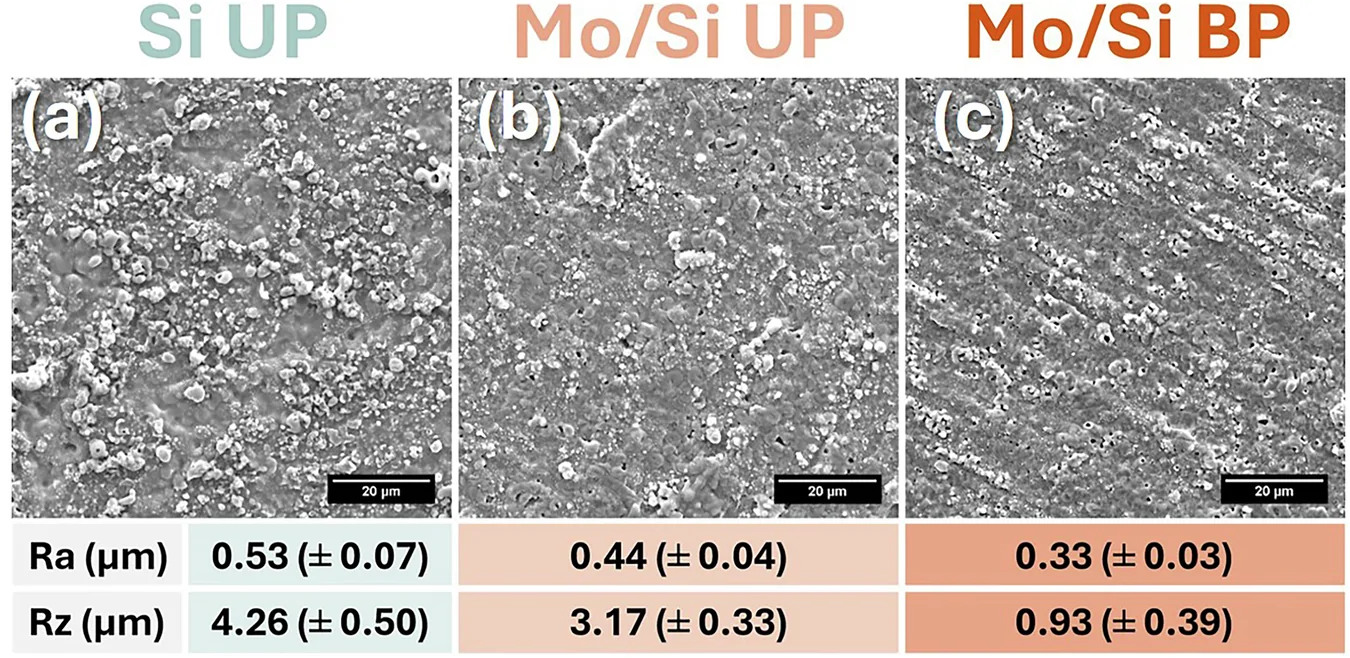
Surface Scanning Electron micrographs. SEM projection of topography for flash-PEO-treated surfaces and corresponding roughness parameters derived by profilometry for **a** Si UP, **b** Mo/Si UP, and **c** Mo/Si BP samples.

SEM micrographs showing the topography of Si UP, Mo/Si UP and Mo/Si BP PEO coatings and surface roughness parameters are presented in Fig. 4. The irregular natured porosity could not be accurately quantified from SEM images and is therefore only described qualitatively. The different PEO treatments result in coatings exhibiting varying morphologies. In the case of the silicate unipolar treatment, a textured surface is exhibited, featuring numerous microscale globular surface features and few open surface pores. In the case of the Mo/Si UP treatment, fewer globular or general protruding surface features are present; however, a higher degree of surface porosity is observed. In the case of the Mo/Si BP-treated samples, even fewer protruding surface features are observed. On the surface of the Mo/Si BP sample, a greater number of open surface pores are present with an approximately smaller average size. Topological measurements for PEO-treated surfaces show agreement with topographic observations, as the roughness (*R*_a_ and *R*_z_) values of Si UP-treated surfaces were greater than those of Mo/Si UP, which in turn exhibited greater roughness than Mo/Si BP-treated samples.

The elemental compositions obtained via EDX for the PEO-treated surfaces are displayed in Table 1. It was observed that whilst aluminium and oxygen were the prominent constituent elements, electrolyte species were incorporated into the surface layer too. Si UP coatings consisted of significant quantities of Si, deposited from the electrolyte. Significant Si uptake was also registered in Mo/Si coatings; however, progressively lower quantities were observed in Mo/Si UP and BP coatings, respectively. This coincides with the observation of decreasing quantities of silicon-rich globules deposited on the surface (Fig. 4).

**Table 1:** Elemental concentrations in atomic percentages as obtained via EDX from a plane view perspective of flash-PEO coatings

at.%	Si UP	Mo/Si UP	Mo/Si BP
Al	35.3	35.9	37.6
O	55.0	55.7	54.8
Si	8.5	7.0	6.55
Mo	–	0.7	0.45
K	0.5	0.5	0.29
Na	0.7	0.2	0.25

Samples treated in the molybdate-containing electrolyte via unipolar and bipolar processes additionally contained 0.72 and 0.45 at.% Mo, respectively. The increased uptake of electrolyte species in the coatings produced via unipolar PEO regimes has been observed elsewhere^13,42^ and similar findings were reported in work comparing the formation of oxide coatings under unipolar and bipolar conditions in tungstate-containing electrolytes^28^. The most likely mechanism for the incorporation of molybdenum into the oxide coating, is through anodic reaction at the electrode surface in the vicinity of plasma discharge channels (1):

1 $${2{\rm{MoO}}}_{4}^{2-}\to {{\rm{MoO}}}_{3}+{4e}^{-}+{{\rm{O}}}_{2}$$

where ${{\rm{MoO}}}_{4}^{2-}$ anions in the electrolyte are transported to the surface under the strong applied electric field. It is hypothesised that the lower uptake of Mo in the oxide during bipolar treatments is a consequence of disruption to the transportation of ${{\rm{Mo}}}_{4}^{2-}$ anions to the surface due to the cyclic switching of the electric field between anodic and cathodic polarisation. However, it is also plausible that other differences in process phenomena, such as the distribution, intensity and energy of surface discharges, could play a role in the incorporation of Mo.

A plausible explanation for the different surface morphologies obtained through different PEO treatments can be found in the processing conditions and electrolyte bath composition. The formation of silicon-rich globules at the surface is hypothesised to be largely facilitated by deposition of electrolytic silica onto the anodic surface. The globular shape of these features suggests their nucleation may occur in the electrolyte, followed by precipitation on the anodic surface. The proposed mechanism for this formation is that local acidification of the electrolyte near the working electrode results in the formation of silica from the silicate electrolyte. Whilst monomeric silica can readily adsorb onto hydrated aluminium oxide surfaces^43^, it is believed that the silicon-rich globule formation is assisted by a concentrated supply of aluminate ions. As such, these globules tend to form at defects (pores, discharge channels or cracks) in the passive layer, which permit strong localised dissolution of the Al substrate under anodic polarisation. Aluminate ions strongly assist the rapid polycondensation of monomeric silica, which can then be deposited on the surface^43^. As a result, Si-rich globule formation effectively acts as a blocking mechanism at defect sites. As such, relatively few pores are observed on the surface of the Si UP-treated alloy. Molybdate anions appear to present a competing blocking mechanism for metal dissolution. In alkaline solutions, ${{\rm{MoO}}}_{4}^{2-}$ could passivate anodic dissolution of Al by adsorbing on the positively charged surface and to a lesser extent by reacting with ${{\rm{AlO}}}_{2}^{-}$ anions in the solution to create a deposited solid product at the surface^44^. If molybdate-induced passivation reactions can occur at the surface quickly, fewer aluminate ions become available in the local electrolyte for silica-rich globule formation. The fact that more globular features are formed by the Mo/Si UP PEO treatment than the corresponding BP treatment can be related to the presence of larger and less controlled discharges during the UP process that create larger defect sites, which can be less readily passivated by inhibitive surface reactions. More aluminium anions will exist in the local electrolyte to induce polycondensation of silicate ions.

**Fig. 5:**
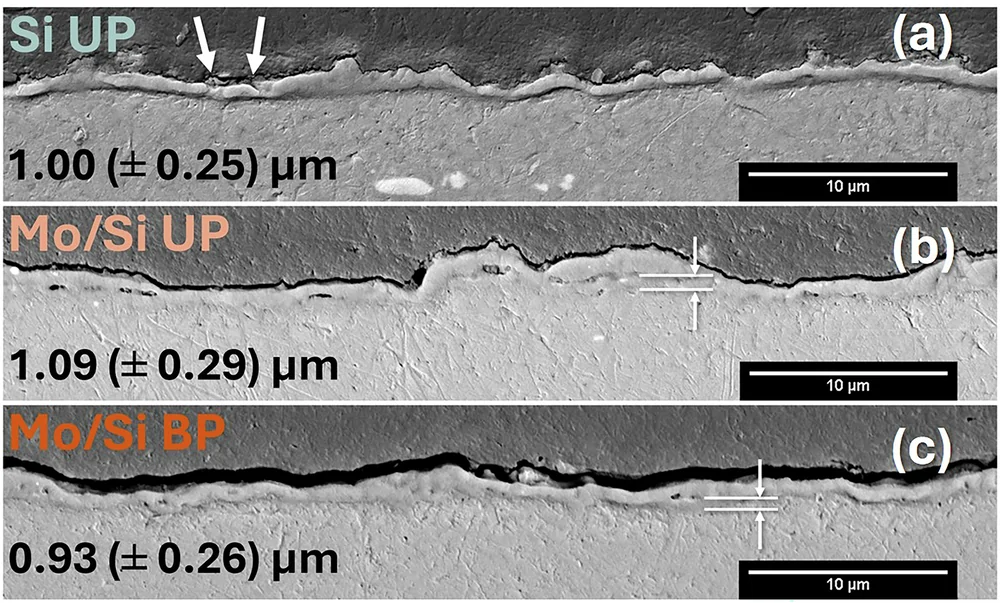
Cross-sectional backscattered electron micrographs. BSE images of the cross sections of oxide layers produced by **a** Si UP, **b** Mo/Si UP and **c** Mo/Si BP flash-PEO treatments. The black overlay gives each coating thickness; the white arrows show defects in the Si UP coating; the white bars and arrows emphasise the continuous barrier layer beneath the porous region in Mo/Si UP and Mo/Si BP coatings.

The cross sections and thicknesses of the oxide coatings produced by each PEO treatment are presented in Fig. 5. It can be observed that relatively dense barrier-layer-type films are formed in each case. All the coatings produced via PEO treatments were of similar approximate thickness (~1 µm). The thickness dimensions of the coatings showed a degree of non-uniformity in each case. In the case of Mo/Si-based treatments, more pores are detected in the oxide coatings; these were generally larger in coatings produced by unipolar processing, which is consistent with plane-view observations. In the case of the Mo treatments, a continuous barrier layer is maintained below any residual porosity. In the case of Si treatments, residual subsurface porosity was less common; however, some defects were observed in the coating.

### Morphology of SAA films

**Fig. 6:**
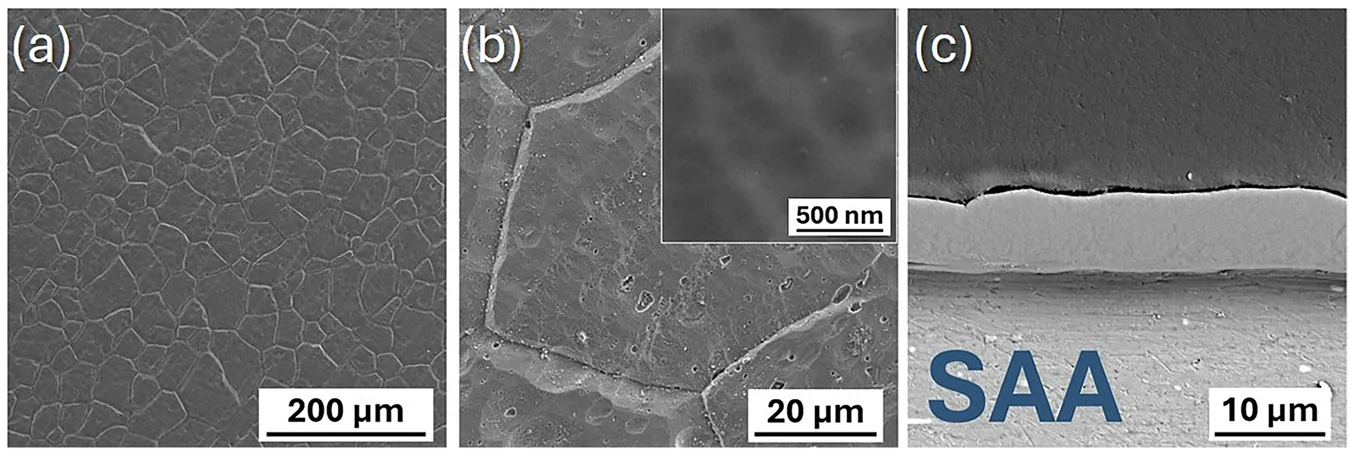
SEM micrographs of SAA-treated surfaces of AA6060-T6 Al alloy. **a** low and **b** medium magnifications. Inset in **b** shows a high-magnification image within the grain. **c** cross-section of the anodic film.

Figure 6 depicts the topography and cross-sectional morphology of the sealed aluminium oxide coating produced by the conventional sulphuric acid anodising treatment utilised in the automotive industry for promoting durability in structurally bonded components. SAA films for structurally bonded components are typically in the range of 2–10 μm, considerably thicker than the flash-PEO coatings investigated in this study. The granular structure observed on the surface results from the anodic film conforming to the grain structure of the underlying Al alloy, which undergoes significant grain boundary attack during the alkaline-etching pre-treatment prior to SAA processing^45^. Whilst a faceted surface is observed on the nanoscale, a dense microstructure rather than the ordered porosity associated with anodic films is observed from both the surface and cross-sectional perspectives. This occurrence of such a dense microstructure can be attributed to the small pore size of the anodic oxide films obtained in the sulphuric acid electrolyte^46^ and the effectiveness of subsequent hydrothermal sealing of the pores. Whilst the eradication of nanoscale porosity by the sealing post-treatment can reduce mechanical interlocking, which can play a key role in adhesive bonding, the procedure is performed to improve the corrosion resistance afforded by the coating. Some larger pores and defects are visible in the coating, which also arise from preferential etching during SAA pre-treatment.

### Surface and coating phase composition

**Fig. 7:**
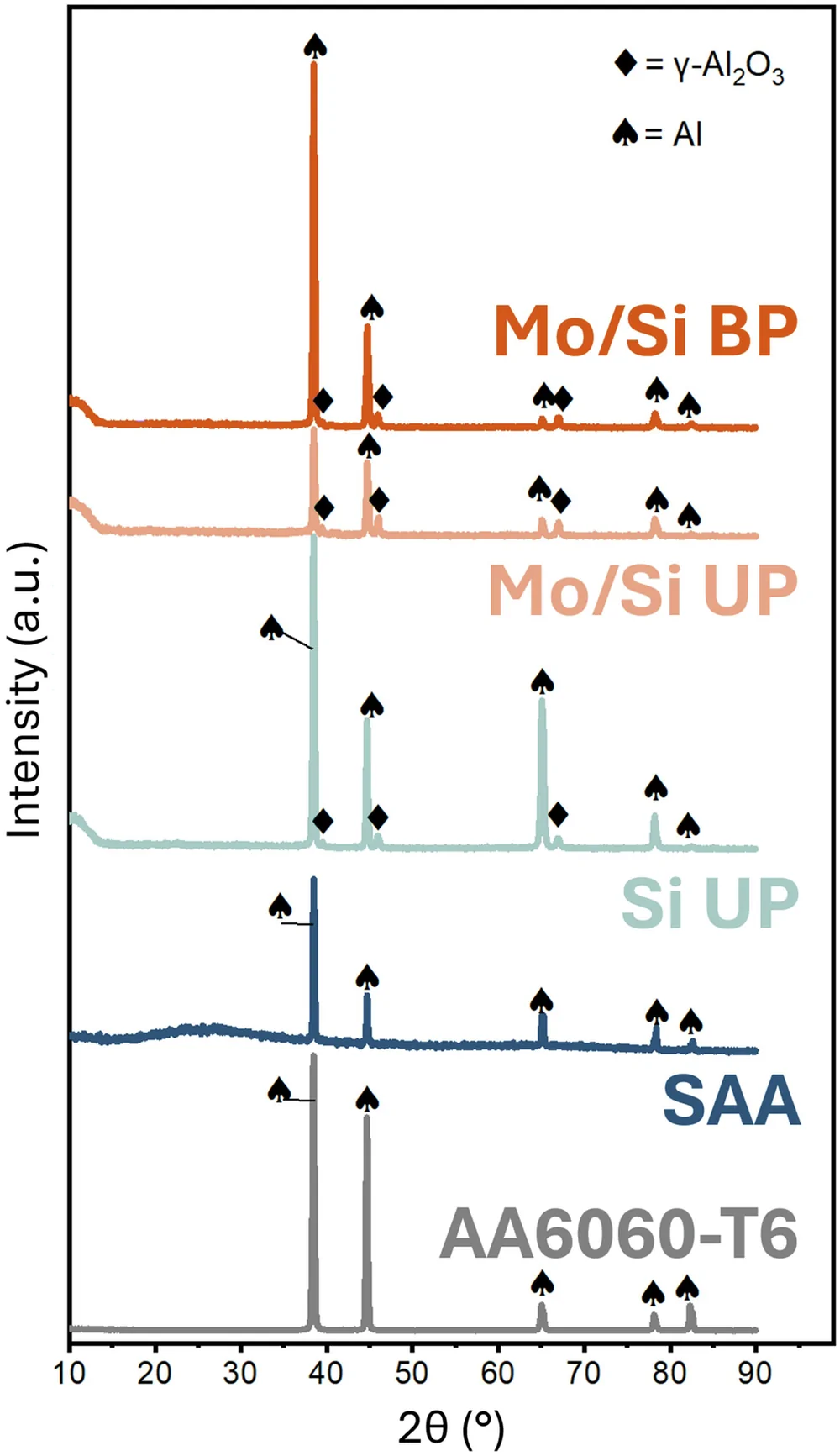
X-ray diffraction spectra. XRD spectra for the studied materials and surface layers.

Figure 7 indicates the presence of γ-alumina phases in the case of Si and Mo/Si PEO coatings and dominant substrate Al peaks due to the thin profile of the coatings. Gamma alumina is a metastable phase typically prevalent in PEO coatings on aluminium due to the fast oxide cooling rates developed on the surface following micro discharge decay; it is particularly dominant in thinner coatings where thermal gradients are steeper, suppressing gamma to alpha transition^47^. No molybdate-containing compounds were observed from the XRD pattern; however, the limited concentrations of 0.45–0.72 at.% Mo in corresponding thin coatings may not be detectable.

Similarly, no crystalline silicate phases could be observed. As both Mo and Si-containing compounds are deposited from the electrolyte, the instance of them residing in an amorphous phase is highly plausible. In the case of the conventional SAA coating, the only crystalline peaks observed can be attributed to the substrate alloy and the only coating feature observed is an amorphous hump in the range of 15°–35° 2*θ*, indicating that the oxide is likely to be fully amorphous.

### Surface composition

The most important phenomena governing adhesive bonding are the occurrence and nature of adsorption of adhesive molecules at the interface^48^. As such, it is important to understand the surface composition governing surface adsorption when assessing the suitability of treated surfaces. XPS was employed to obtain surface-sensitive chemical characteristics of the outermost region of the treated surfaces. Survey XPS scans of the PEO and SAA coatings are depicted in Fig. S2, with quantitative data presented in Table 2, respectively. Significant carbon concentration is observed on all samples, which is attributed to ‘adventitious’ carbon contamination^49^. N and Ca contributions are associated with ambient exposure. The quantification results obtained suggest that the surface chemistry of the different PEO coatings is very similar. In each case, the surface composition is dominated by the presence of oxygen with significant quantities of Si and Al, indicating the likely existence of aluminium oxide/hydroxides, silica and/or aluminosilicate compounds at the surface.

**Table 2:** XPS quantification table for surface composition of the studied coatings (at. %)

at. %	O	Si	Al	Mo	K	Na	C	N	Ca	F	S
Quant. Peak	**1s**	**2p**	**2p**	**3d**	**2p**	**1s**	**1s**	**1s**	**2p**	**1s**	**2p**
Si UP	43.1	15.4	8.5	–	2.1	1.3	23.8	2.5	3.3	–	–
Mo/Si UP	40.8	13.1	6.4	0.3	2.1	0.3	31.2	5.4	0.4	–	–
Mo/Si BP	43.1	13.1	7.1	0.3	1.4	0.2	29.8	4.3	0.7	–	–
SAA	37.7	–	15.2	–	–	0.3	40.0	1.9	0.4	0.8	3.7

Whilst the concentration of O, Al and Si elements detected via XPS varied to a degree between PEO-treated surfaces, the concentrations fell within a similar range. Trace amounts of electrolyte cations, K and Na, are observed at the surfaces of all PEO coatings, whilst Mo is present in small quantities at the surface for Mo/Si samples. Quantification of the SAA surface revealed a surface composition consisting of oxygen and a comparatively higher concentration of aluminium which could be attributed to a primary surface composition of aluminium oxides and hydroxides alongside trace quantities of F, S and Na, which can be attributed to small quantities of surface remnants from different processing steps in the manufacturing history of the treated surface.

**Fig. 8:**
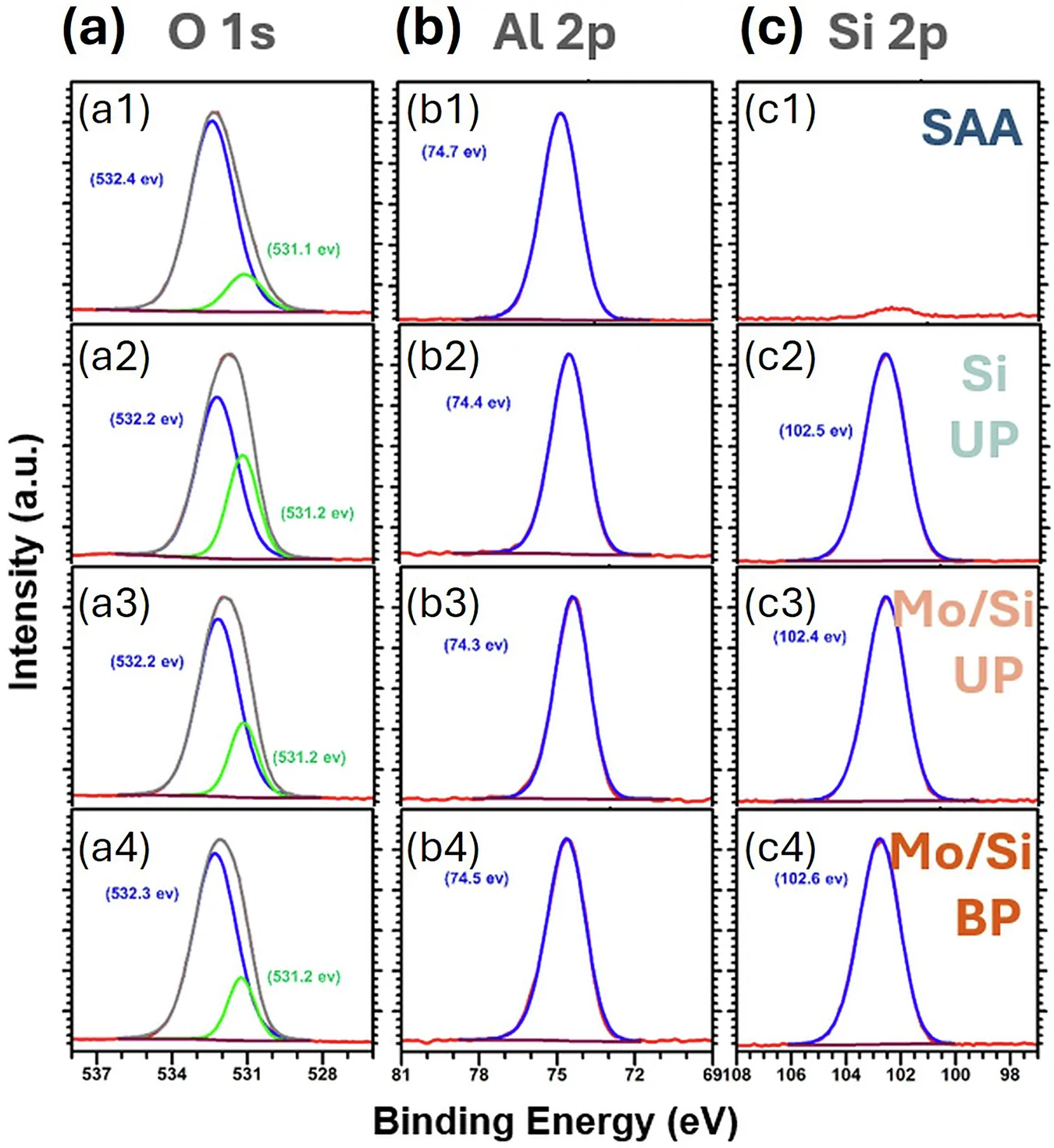
High resolution XPS spectra of the studied coatings. **a** O 1*s*, **b** Al 2*p* and **c** Si 2*p* regions. (a1), (b1) and (c1)—SAA film. (a2), (b2) and (c2)—Si UP; (a3), (b3) and (c3)—Mo/Si UP; (a4), (b4) and (c4)—Mo/Si BP flash PEO coatings.

Figure 8 compares high-resolution XPS scans of O 1*s*, Si 2*p* and Al 2*p* peaks obtained from the treated surfaces. The O1*s* peaks for Si and both Mo/Si PEO coatings were deconvoluted into two components. A larger peak in the range of 532.2–532.3 eV represents both O–Si bonds and hydroxyls that are present due to hydration of the oxide surface^50^. The Si–O contribution corresponds with Si 2*p* peaks observed at 102.4–102.6 eV on PEO-treated surfaces, indicating the presence of silicate compounds, which could indicate SiO_2_^51^ or aluminosilicate phases, including halloysite and kaolinite, which are favoured compounds when monomeric silica precipitates on γ-alumina^43^. A second, smaller O1*s* peak recorded at 531.2 eV corresponds to Al–O bonding and is indicative of aluminosilicate compounds or aluminium oxide^52,53^. The Al 2*p* peaks situated at 74.3–74.5 eV for PEO treated surfaces correspond to γ-alumina and aluminosilicate phases^15,54^. The O1*s* peak at 531.2 eV also corresponds to Mo–O bonding^55^.

**Fig. 9:**
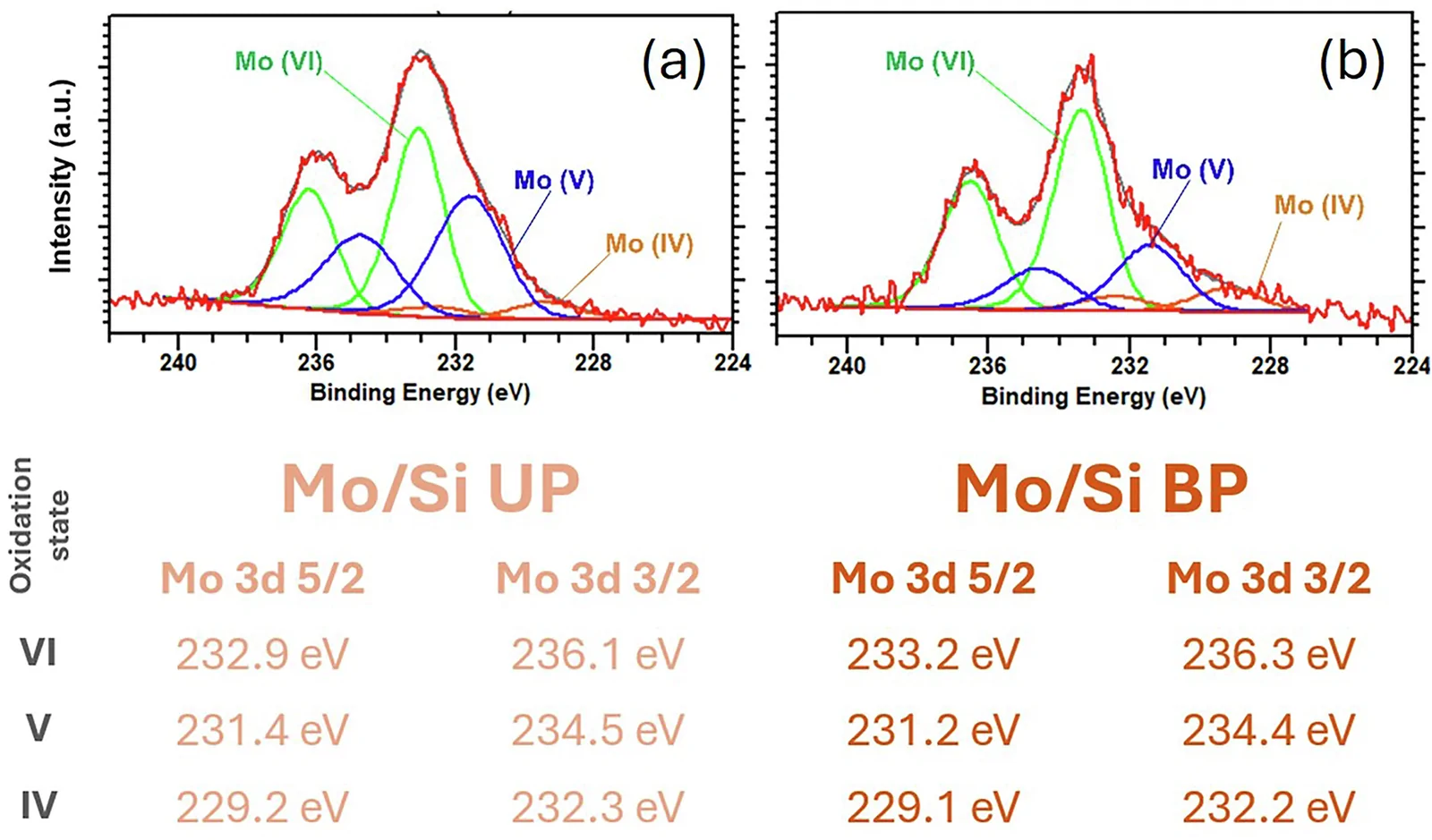
High-resolution Mo 3d photoelectron spectra of flash-PEO coatings. **a** Mo/Si UP **b** Mo/Si BP flash-PEO coatings. Deconvoluted peak binding energies.

Deconvoluted spectra for Mo 3*d* peaks obtained at the surface of Mo/Si UP and Mo/Si BP treated samples are displayed in Fig. 9. The position of the deconvoluted peaks corresponds to Mo(VI), Mo(V) and Mo(IV)^56,57^. In both coatings, molybdenum is most abundant in the 6+ oxidation state, which aligns with the proposed anodic reaction for incorporation of molybdenum into the coating in the form of MoO_3_. The presence of Mo(V) and, to a lesser extent, Mo(IV) at the surface is indicative of mixed valence compounds involved in redox processes that may be influential on corrosion-resistant behaviour at the surface of Al^58,59^. For SAA-treated samples, the O1*s* peak deconvoluted to two peaks at 531.1 and 532.4 eV which are attributed to Al–O–Al bonding in aluminium oxide and Al–O–H bonding in structural hydroxyls in the sealed surface^60,61^. The Al 2*p* peak at 74.7 eV is consistent with Al–O bonding in aluminium hydroxides^60,62^.

### Surface wettability

**Fig. 10:**
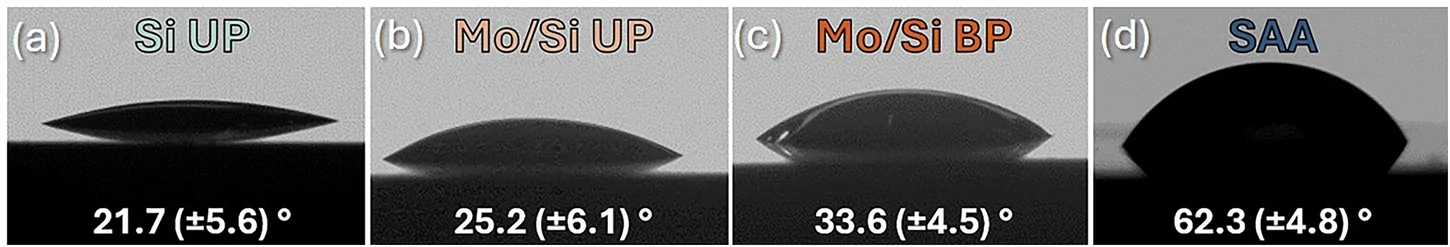
Contact angle measurements for coated AA6060-T6 samples. **a** Si UP, **b** Mo/Si UP, **c** Mo/Si BP, and **d** SAA.

Allowing for complete wetting of the surface by the adhesive is important for the establishment of a strong interface for high adhesive strength and durability^4^. Results of water droplet contact angle measurements carried out on treated samples are presented in Fig. 10, indicating that all treatments provided hydrophilic surfaces. PEO coatings were observed to exhibit greater wettability than conventional sealed SAA-treated samples. Of the PEO-treated samples, the Si UP coating exhibited the lowest average contact angle (21.7°) and the Mo/Si BP (33.6°) surface exhibited the highest. Aside from the presence of molybdenum oxides that made up a small percentage of the surface composition in the case of Mo/Si treatments, the surface chemical composition was similar across the PEO coatings, which had similar quantities of O, Si and Al. Whilst small quantities of molybdenum oxide may have some influence on surface hydrophilicity, molybdenum oxides are not expected to have a significant detrimental effect and have been incorporated into hydrophilic conversion coatings^63^. It is hypothesised that geometric factors are dominant in dictating the surface wettability of PEO-treated surfaces within this study, where a direct relationship between the surface roughness of PEO-treated samples and surface contact angle measurement was observed. Surface roughness has been cited as a dominant factor influencing the hydrophobicity of PEO coatings of different compositions in previous investigations^18^.

### Open circuit potential evolution

**Fig. 11:**
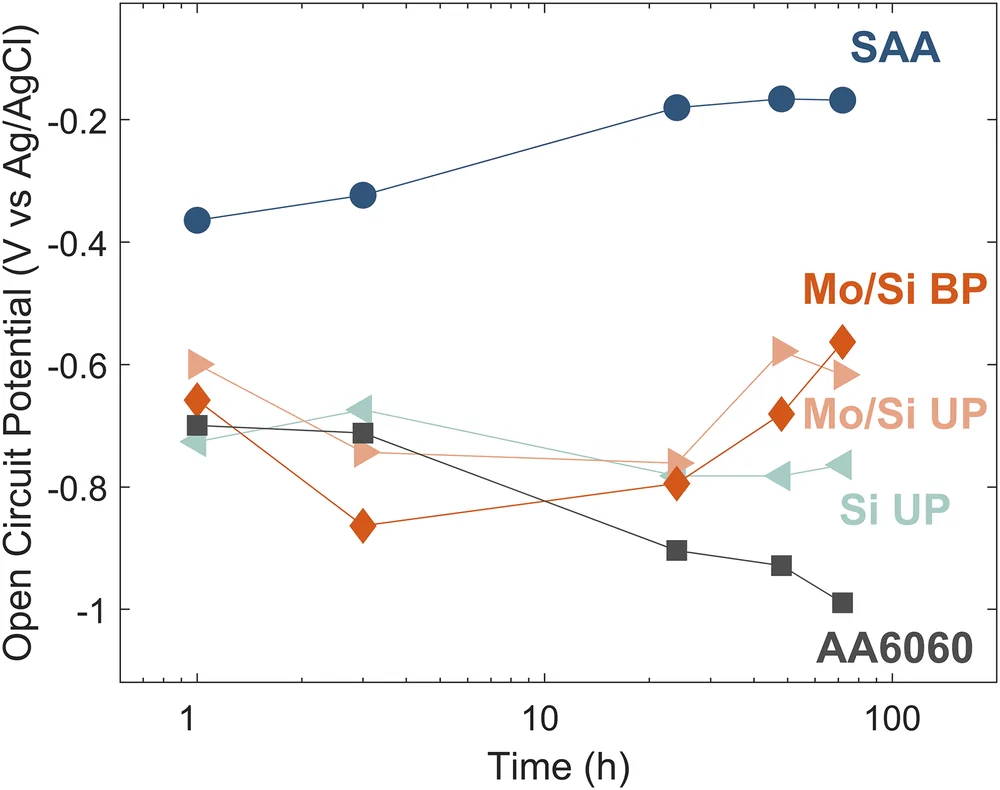
Open circuit potential. Evolution of open circuit potential vs. Ag/AgCl /KCl saturated electrode for the studied flash-PEO coatings over 72 h of immersion in 3.5% NaCl.

The OCP of the investigated surface treatments was measured periodically over a 72-h exposure period to provide an insight into the evolution of corrosion processes at the surface (Fig. 11). The OCP can indicate the impetus required for the surface to undergo anodic or cathodic reactions. More negative OCP values mean lower anodic overpotentials are required to drive the oxidation reaction on the surface, which can thus be more susceptible to corrosion. The untreated AA6060-T6 sample exhibited a comparatively stable OCP within the initial 3 h of exposure; however, it showed the most negative OCP after 24 h, and over the 72-h measurement period showed the greatest negative shift in OCP of all measured surfaces, dropping from −0.70 to −0.99 V (vs. Ag/AgCl sat). This indicates the dominance of the anodic half-reaction over the cathodic half-reaction on the untreated surface.

The Si UP PEO treated surface saw a marginal increase in OCP from −0.72 to −0.67 V in the initial 3 h of exposure before dropping and settling at −0.78 V between 48 and 72 h. This may be because of time-dependent electrolyte ingress into the coating between 3 and 48 h, leading to increased surface/electrolyte interactions. In the case of the Mo/Si BP PEO coating, a dramatic decrease in OCP is observed in the first 3 h (from −0.67 V after 1 h to −0.86 V at 3 h) before observing a gradual increase to more noble values of −0.68 and −0.56 V at 48 and 72 h, respectively. In the case of the Mo/Si UP PEO sample, a less drastic decrease in OCP is observed in the initial 3 hours of exposure before an increase to more noble values is observed between 24 and 48 h. It was observed that the OCP showed a small reduction between 48 and 72 h. It is hypothesised that the initial fall in OCP values in the Mo-containing coated surfaces could be due to more penetrable surface morphologies being more quickly impregnated by the electrolyte. However, the effect of molybdate species leaching from the coating into the local electrolyte during the initial attack of the coating on the local pH conditions could also be influential. Aqueous molybdate inhibitors have been reported to show such initial effects on the OCP^59^. The comparative increase towards more positive OCP values over 72 h in PEO treatments that contained Mo compounds could be a result of improved passivation. In such a case, initial corrosion products may contribute to inhibition of corrosion or pit nucleation at weak points in the oxide barrier^58,64^. The OCP measurements recorded for the sealed SAA-treated alloy suggest that excellent corrosion protection is afforded by this treatment, with the OCP values being considerably more noble than those obtained for the untreated alloy. An initial increase in OCP is observed over the first 24 h, indicating increased surface passivation and a very stable response at the nobler potential of −0.18 V thereafter, indicating stability of the sealed anodic oxide. This is consistent with findings in literature^65^ where researchers have suggested that such stable and noble OCP behaviour in anodised coatings is dependent on the quality of the sealing post-treatment.

### Evolution of impedance spectra

**Fig. 12:**
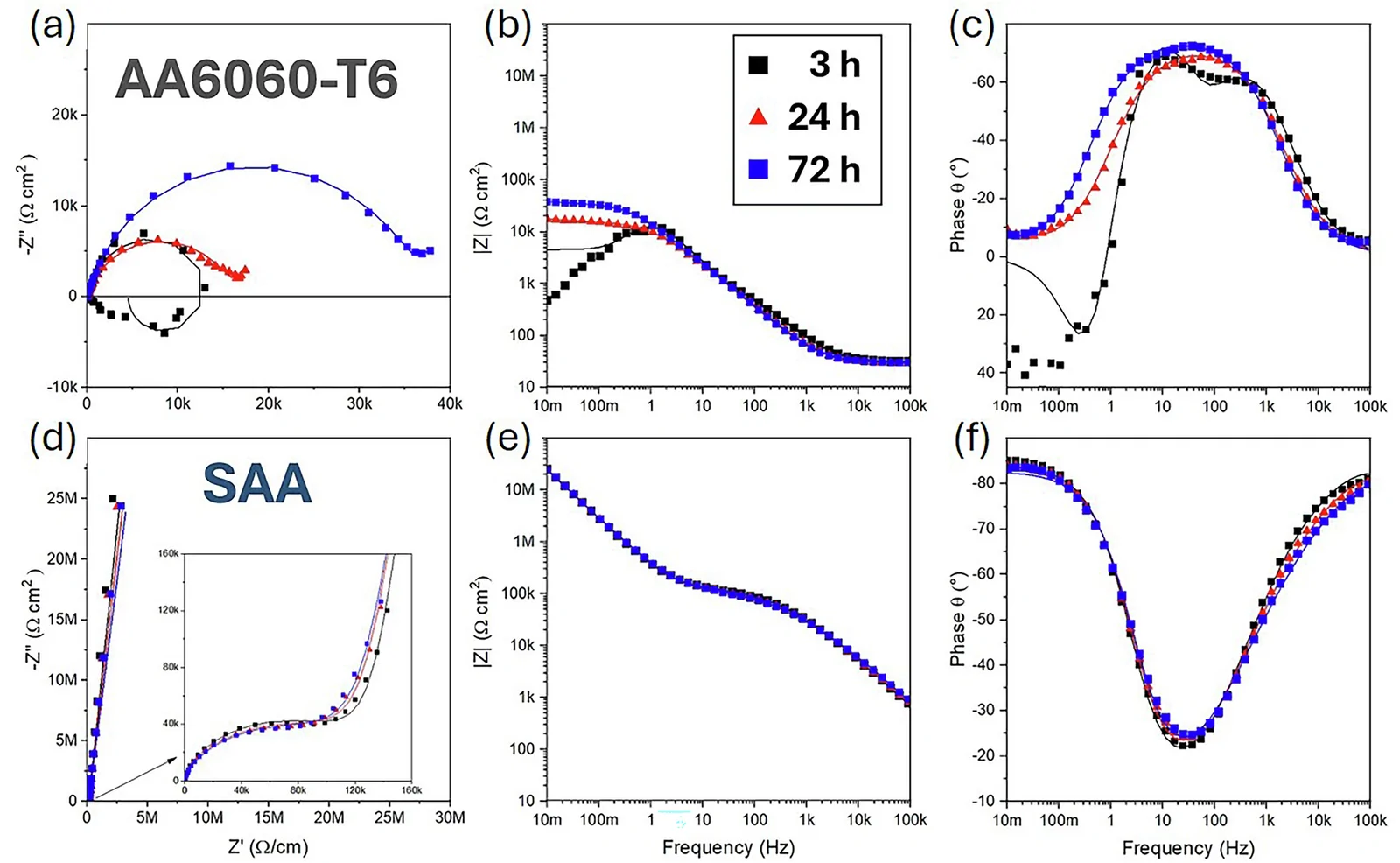
Electrochemical impedance spectra for untreated and SAA treated AA6060-T6 after 3, 24 and 72 h of exposure to 3.5 wt% NaCl. EIS spectra for **a**–**c** untreated AA6060-T6 alloy and **d**–**f** sealed SAA coating after 3, 24 and 72 h of exposure to 3.5 wt% NaCl: **a**, **d** Nyquist, **b**, **e** Bode impedance and **c**, **f** Bode phase angle plots.

**Fig. 13:**
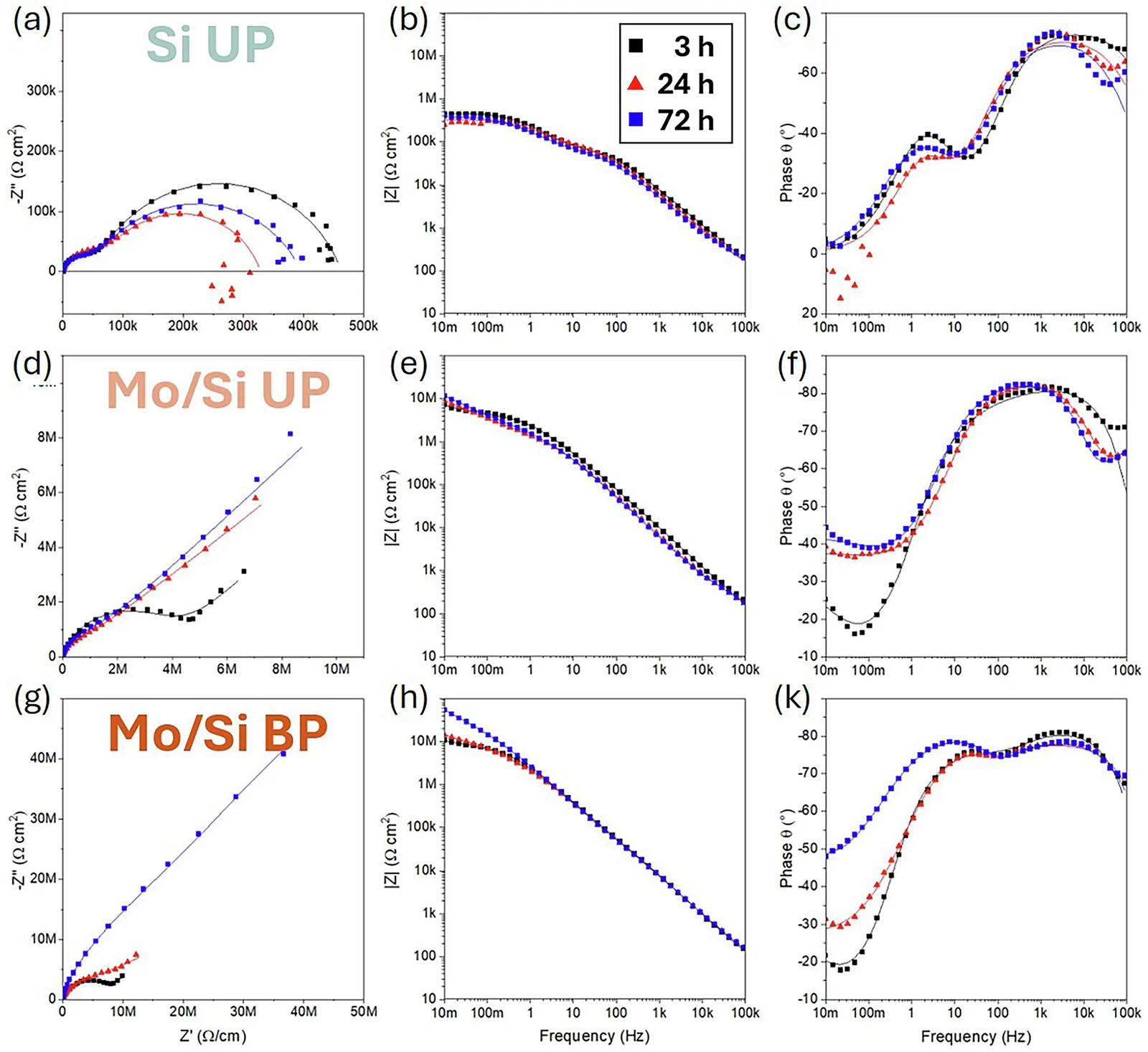
EIS spectra for flash-PEO-coated AA6060-T6 after 3, 24 and 72 h of exposure to 3.5 wt% NaCl. **a**–**c** Si UP, **d**–**f** Mo/Si UP, **g**–**k** Mo/Si BP treatments. **a**, **d** and **g** Nyquist, **b**, **e** and **h** Bode impedance, and **c**, **f** and **k** Bode phase angle plots.

**Fig. 14:**
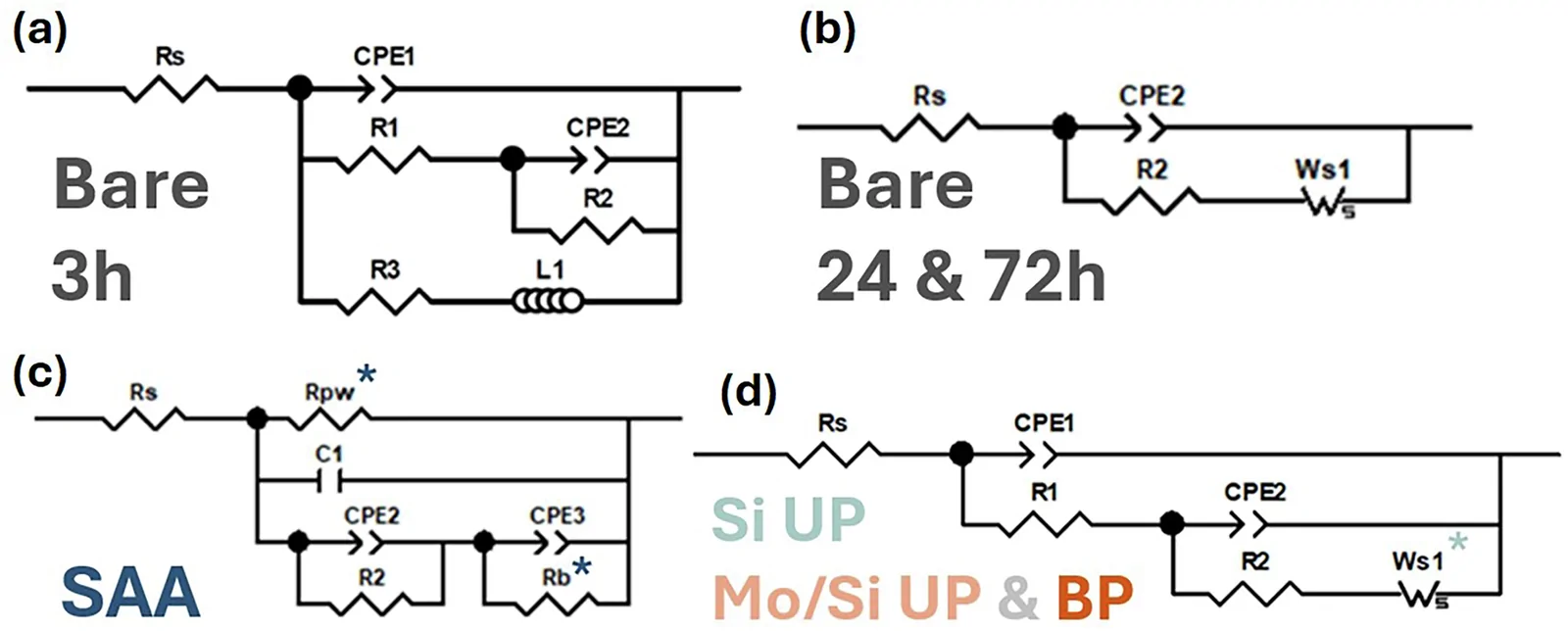
Equivalent circuits used to fit EIS data for each class of samples. **a** Bare AA6060-T6 after 3 h and **b** after 24 and 72 h. **c** Sealed SAA, **d** Si UP and Mo/Si UP and Mo/Si BP-treated samples. Asterisks mark additional constraints in the fitting procedures to some particular circuit elements: for SAA *R*_b_ and *R*_pw_ were omitted (considered as infinitely large impedance); for Si UP, Ws1 was omitted (considered as zero impedance amplitude).

Electrochemical impedance spectroscopy was utilised to obtain a better understanding of corrosion processes occurring at the sample surfaces in an aerated 3.5% NaCl solution after 3, 24 and 72 h of exposure. Nyquist and Bode plots for bare and SAA-treated AA6060-T6 samples are displayed in Fig. 12, and for flash-PEO in Fig. 13. The equivalent circuits used to fit the data in each case are displayed in Fig. 14. In the equivalent circuits, Rs represents the solution resistance, whilst constant phase elements (CPEs) were used instead of capacitors in many cases to represent the non-ideal capacitive behaviour of the coating components. All impedances are area-normalised (e.g. Ω cm²) and all values of EIS fitted parameters are provided in Supplementary Materials, Table S1.

The EIS spectra for bare AA6060-T6 at 3 h show substantial noise at low frequencies, likely caused by unstable OCP behaviour. An inductive loop was identified at low frequencies, which can be associated with the active anodic dissolution of aluminium^58,66^. Two additional time constants are observed; the first of these, CPE2 and *R*2, can be attributed to the electric double-layer capacitance and charge-transfer resistance at the naturally forming passive oxide. The second time constant at higher frequencies, CPE1 and *R*1, is not distinguishable at longer exposure times and is likely attributable to charge transfer resistance provided by unstable corrosion products, the electrochemical behaviour of which evolves over time. The charge transfer resistance (*R*2) was observed to increase over time up to 72 h, which can be attributed to increased passivation afforded by the initial oxidation upon exposure to the electrolyte. At 24 and 72 h, no inductive behaviour is observed at low frequency; instead, what appears to be a diffusion-controlled impedance component represented by a Warburg element (WS1) is present, which is characteristic of limitations in mass transfer caused by initial corrosion products^67^.

In the case of the SAA-treated samples, comparatively little evolution in impedance response was observed over the 72 h of immersion. This indicates stable corrosion behaviour with little change to the surface structure and composition over the studied period. Impedance response of the sealed SAA treated surface was fitted using the corresponding equivalent circuit presented in Fig. 14, with the further constraint of assuming *R*_b_ ≈ *R*_pw_ ≈ ∞. At higher frequencies, CPE2 and *R*2 represent the sealed region of pores, whilst C1 represents the capacitance of the pore wall. At lower frequency, highly capacitive behaviour is observed signified by the close-to-vertical -*Z*” vs. *Z*’ relationship. This region was ascribed to the inner dense barrier layer of the anodic coating, which was modelled using a single constant phase element (CPE3). The *R*_b_ and *R*_pw_ components, representing the resistive elements of the barrier layer and of the pore walls, respectively, have been constrained to infinitely large impedances, which is equivalent to omitting them from the model circuit. This is justified by the highly capacitive behaviour of the impedance at the selected frequency range, resulting in only a lower-bound value being extractable from the fitting (*R*_b_, *R*_pw_ > ~10^9^ Ω cm^2^). Such simplifications have often been inferred by authors modelling the barrier layer impedance response of anodic oxide films^68,69^. This almost ideal, time-invariant capacitive behaviour is indicative of a high-quality-defect-free dielectric coating and in line with expectations from a relatively thick, dense and sealed ceramic coating.

EIS data and fits for the investigated PEO-treated surfaces are displayed in the form of Nyquist and Bode plots in Fig. 13. In each case, two capacitive time constants were modelled with CPE components. The higher frequency loop (CPE1/*R*1) represents the resistance and capacitance associated with the bulk barrier layer and the lower frequency loop (CPE2/*R*2) represents the charge transfer resistance and double-layer capacitance at the oxide/substrate interface. In the case of Mo/Si UP and Mo/Si BP coatings, an additional Warburg component (WS1) represents a diffusion-controlled process attributed to limited mass transfer^70^. This process and diffusion-like behaviour are absent and therefore omitted in Si UP data fitting. The difference in magnitude of the charge-transfer resistive components *R*1 and *R*2 for this coating is markedly smaller than in other PEO coatings, demonstrated by the observable double arch on the corresponding Nyquist plots.

**Fig. 15:**
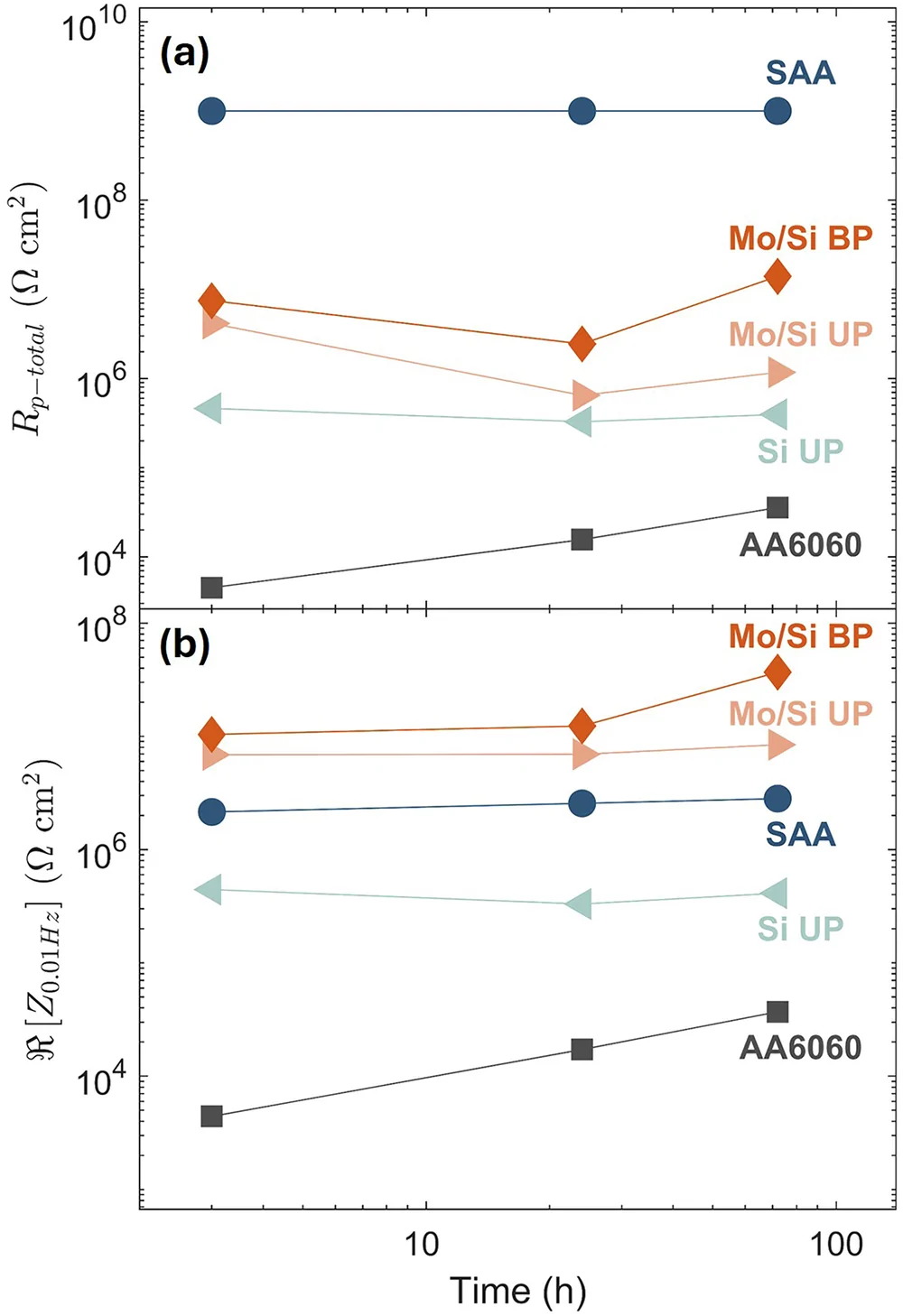
Evolution of impedance characteristics for the studied samples after 3, 24 and 72 h of exposure to 3.5 wt% NaCl. **a** effective total charge transfer resistance (*R*_p-total_) **b** real part of impedance *Z* at the lowest frequency probed (ℜ[*Z*_0.01 Hz_]). Note, for SAA *R*_p-total_ only denotes a lower-bound estimate, whereas for Mo/Si UP and Mo/Si BP samples, ℜ[Z_0.01 Hz_] is significantly inflated by diffusion components.

Two additional quantities were derived and are presented in Fig. 15, for more direct comparison of surfaces for which EIS data were fitted using different equivalent circuits. *R*_p-total_ is the effective total charge transfer resistance calculated from the fitted parameter values of each circuit. ℜ[*Z*_0.01 Hz_] is the real part of complex impedance *Z* at the lowest frequency in the probed range. The value of ℜ[*Z*_0.01 Hz_] can be inflated by diffusive processes and thus cannot be directly linked to corrosion resistance, but it does present the advantage of being agnostic to the fitting model used and can shed light on trends. The Si UP sample showed a small initial decline in *R*_p-total_ between 3 and 24-h measurements, before increasing again slightly from 24 to 72 h. This suggests that initial degradation of the Si UP PEO coating likely occurs, which leads to the formation of relatively dense corrosion products, which then increase passivity.

After the initial 3 h measurements, both UP and BP Mo/Si-treated samples exhibited a total charge-transfer resistance *R*_p-total_, an order of magnitude greater than that of the Si UP coating. The magnitude of total charge-transfer resistance for Mo/Si UP and Mo/Si BP-treated surfaces followed a similar trend to Si UP-treated surfaces, exhibiting an initial drop between 3 and 24 h followed by an increase in magnitude up to 72 h, which could be attributed to shielding from corrosion by the initial passivation reactions. It was observed that the bipolar treated surface exhibited a higher *R*_p-total_ value over the observation period than the unipolar Mo/Si-treated surface. Mo/Si UP and Mo/Si BP PEO coatings demonstrated significantly higher interfacial resistance (*R*2) values and notably smaller interfacial capacitance (CPE2) compared to Si UP-treated surfaces, indicating higher charge transfer resistance at the interface due to a thicker dielectric region. This suggests that aside from the polarisation parameters, the inclusion of molybdate ions in the electrolyte has a direct or synergistic role in the formation of a more corrosion-resistant barrier. The bulk layer impedance (*R*1) contributed to a lower proportion of the overall charge-transfer resistance in the case of Mo/Si coatings, but was still significant in the case of bipolar processing (greater than Si UP). However, for Mo/Si UP treatments, this was observed to be far smaller in magnitude. The *R*1/CPE 1 time constant is not easily distinguishable at 24–72 h and was fitted with a value several orders of magnitude lower than the bulk *R*1 value attributed to the Mo/Si BP and Si UP coatings. From the characterisation of the physical and chemical features of the coating, it cannot be clearly determined why this component is significantly lower in the case of the Mo/Si UP coating.

Whilst *R*_p-total_ and ℜ[Z_0.01 Hz_] are relatively close to one another for Si UP-treated samples, there is a large discrepancy between these quantities in the case of Mo/Si coatings, as the low-frequency response for both UP and BP-treated samples is additionally influenced by the diffusion-controlled mass transfer behaviour represented by WS1. This is observable in corresponding Nyquist plots as the close to 45° line at the low-frequency end of each spectrum. For these samples, the magnitude of mass-transfer impedance Ws-R rises incrementally over 72 h of exposure. The resistance to diffusion may be due to the formation of products in the coating or metal interface, which can obstruct species diffusion. For Mo/Si BP, this increase is significantly more pronounced. Moreover, for the same Mo/Si BP coating, the Warburg diffusive time constant Ws-T shows a strong decreasing trend, with the 72-h value over an order of magnitude smaller than after 3 h, suggesting shrinking characteristic diffusion lengths as exposure is prolonged. This trend can be linked with the more noble OCP behaviour with exposure time and with the presence of Mo in different oxidation states shown via XPS to suggest that the smaller, more evenly distributed pores of Mo/Si BP are partially sealed by the products of corrosion, starting from the top of the coating. This would indeed lead to shallower diffusion with higher resistance to diffusion and result in the high and heavily diffusion-inflated ℜ[*Z*_0.01 Hz_] compatible with but independent from the increasing charge-transfer resistance.

The EIS results suggest that whilst all PEO samples consistently exhibit significantly higher polarisation resistance than bare AA6060-T6, the Mo/Si BP sample shows the highest level of corrosion protection. Whilst at 72 h, Mo/Si BP exhibits a greater ℜ[Z_0.01 Hz_] value than the sealed SAA, this was largely due to the contribution of the diffusion-controlled mechanism, the effectiveness of which in sustaining corrosion protection over a longer term is uncertain. Compared to the evolving electrochemical behaviour of PEO coatings, SAA-treated samples exhibited highly stable impedance behaviour as should be expected from sealed coatings.

### Adhesive bond strength

**Fig. 16:**
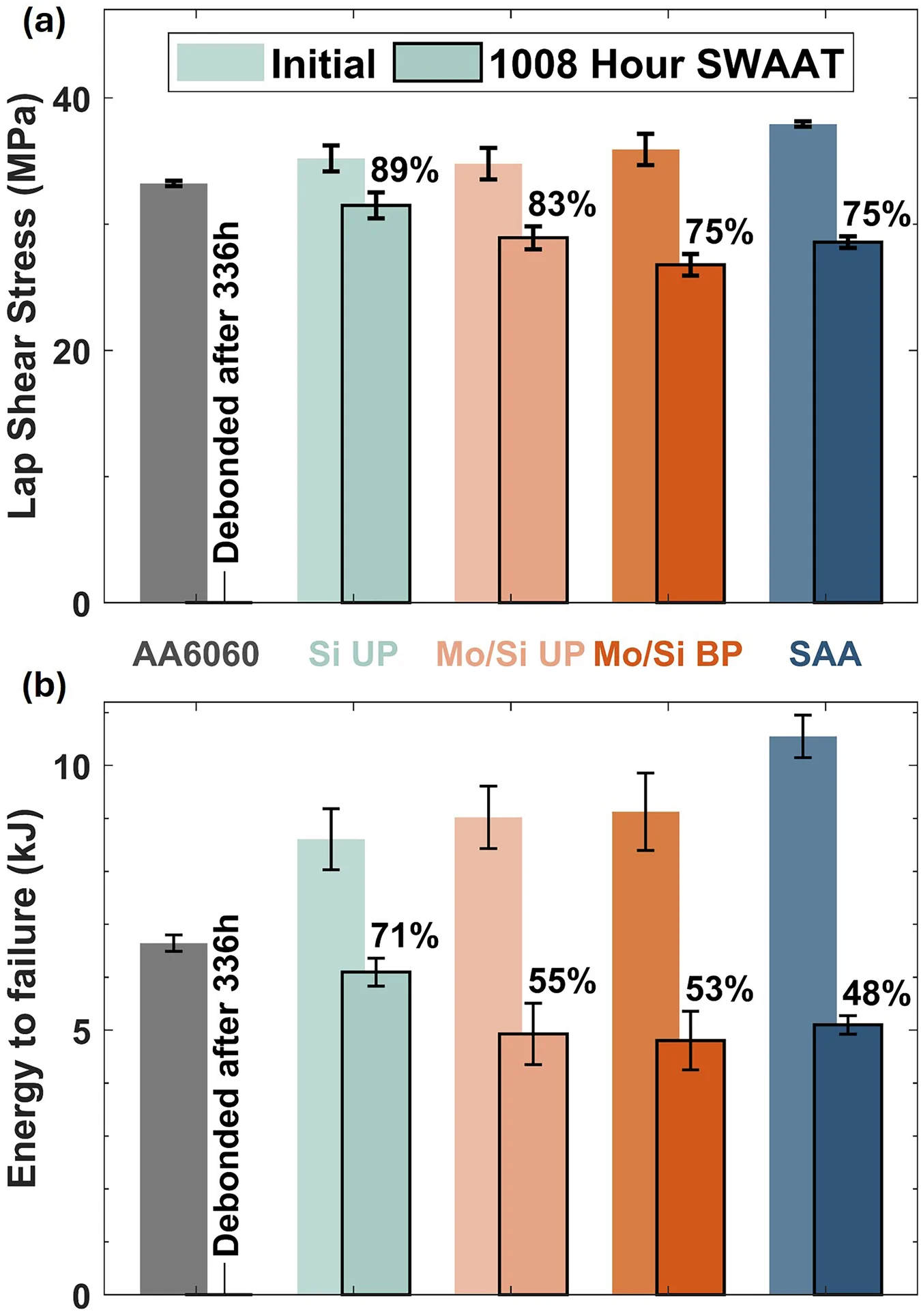
Lap shear test results. Stress (**a**) and energy to failure (**b**) values obtained in lap shear testing before and after 1008 h of SWAAT exposure of adhesively bonded AA6060-T6 samples in uncoated, flash-PEO-treated and sealed SAA conditions.

Lap shear joints were tested to failure, following curing and cooling to room temperature, to analyse the maximum bond strength of joints in the ‘as-new’ condition. The ultimate lap shear strengths obtained for ‘as-new’ PEO, SAA and untreated AA6060 coupons are displayed in Fig. 16 alongside the energy to failure (right) calculated from the force extension data plotted in Supplementary Materials, Fig. S3. Whilst the industry standard SAA surface treatment failed at the highest shear stress (37.9 ± 0.2 MPa), PEO-treated samples failed at comparable lap shear strength, just below this value. Si UP-treated samples failed at 35.2 + ( ± 1.0) MPa, whilst Mo/Si UP and BP samples failed at an average lap shear stress of 34.8 (±1.2) and 35.9 (±1.2) MPa, respectively. Untreated AA6060-T6 samples withstood high loading under unaged conditions, failing at an average lap shear stress of 33.2 (±0.2) MPa. It was observed that all lap shear joints, including those of untreated samples, failed at high loading, surpassing the benchmark load and energy values of 7.1 kN and 5 J, respectively, adopted as a pass/fail threshold in the automotive industry when testing sealed SAA for structural bonding under the same test conditions^71^.

**Fig. 17:**
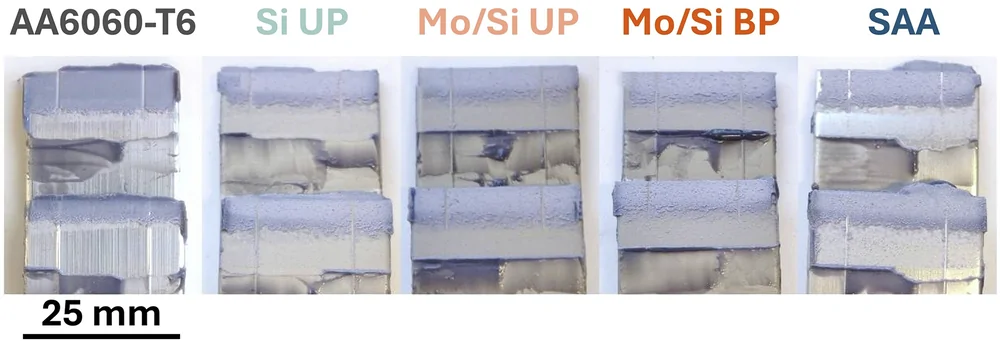
Fracture surfaces of unaged joints. Fracture surfaces obtained before 1008 h SWAAT tests of adhesively bonded AA6060-T6 samples in uncoated, flash-PEO-treated and sealed SAA condition.

**Fig. 18:**
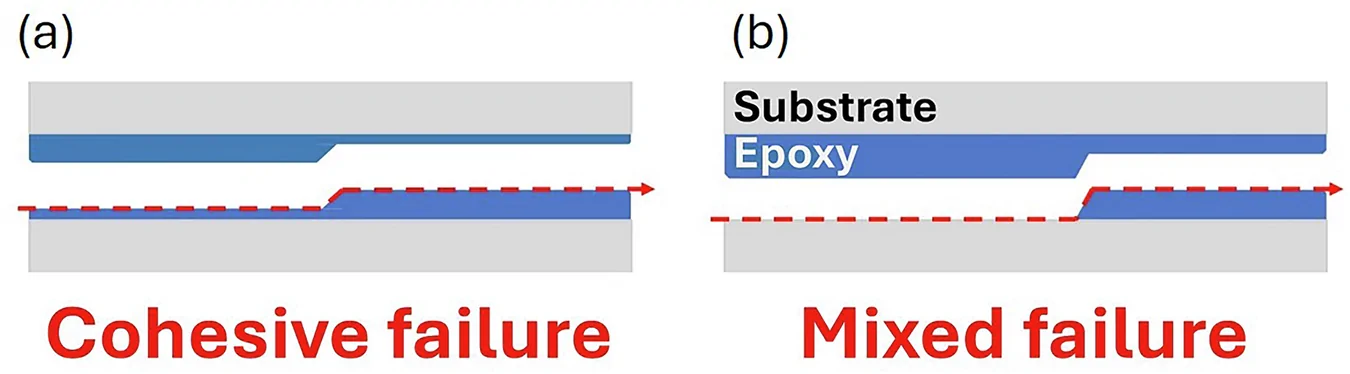
Adhesive bond failure modes. **a** Is 50/50 near-surface cohesive failure, as observed in the case of treated samples. **b** Is a mixed failure containing regions of interfacial adhesive failure and failure within the epoxy, as observed in the case of untreated AA6060-T6. Dashed line is indicative of the fracture path.

The mode of failure and the region in which failure occurs are as important in assessing the suitability of a surface treatment for bonding applications as the maximum obtained strength of the joint. For a surface treatment to be considered suitable, it is preferred that failure occurs cohesively within the adhesive phase and not at the adhesive/adherent interface nor cohesively within either coating or substrate. Figure 17 shows that all treated joints exhibit similar fractures, displaying the type of 50/50 fracture surface observed in our previous study^11^. The ‘50/50 fracture’ can be summarised as deceptive close-to-surface cohesive failure within the adhesive, occurring on different sides of the adhesive region in the opposing halves of the joint. This is caused by symmetrical loading conditions imposed on the joint under tensile loading. This cohesive failure fracture behaviour is schematically demonstrated in Fig. 18.

Whilst fracture surfaces obtained for AA6060-T6 similarly resulted in epoxy left on opposing faces of the joint (albeit in varying proportions), under closer investigation, it was evident that in this case, the majority of the ‘bare region’ represented a clean alloy surface, indicating complete removal of epoxy material (EDX evidence provided in Supplementary Materials, Figs. S4 and S5). This indicates that partial interfacial adhesive failure, schematised as mixed failure in Fig. 18, occurred on untreated samples. It indicates a weaker interface between adherent and adhesive, which can be attributed to the reduced mechanical interlocking effect without surface pre-treatment applied to the Al alloy.

The Mo/Si and Si PEO-treated samples investigated withstood marginally higher loading than the best-performing coatings produced under potentiostatic PEO in our previous studies^11^. PEO-treated samples also showed significantly higher ultimate lap shear strengths than adhesive bonded samples treated via PEO^21–24,72^ and anodising^73,74^ on various metal substrates and under varying lap shear test conditions in the literature. The results suggest that flash-type PEO treatments can be considered a promising technique for the provision of topological and chemical modification of aluminium for adhesive bonding.

### Adhesive bond durability

To examine the effect of the surface treatments on the bond durability of adhesively bonded AA6060-T6, lap shear joints were subjected to accelerated corrosive conditions in the form of 1008 h SWAAT exposure prior to lap shear testing to failure under identical loading conditions as bonded coupons in the ‘unaged’ condition. The ASTM G85 A3 SWAAT test presents a much harsher corrosive environment than likely in-service conditions of such materials; however, the harsh cyclic conditions produced by the acidic medium at high humidity and temperature provide an incredibly stern test for the bonded samples, which can be carried out in a matter of weeks or months. This has made the test a valuable tool in the automotive industry for verifying the durability of components^6,75^.

**Fig. 19:**
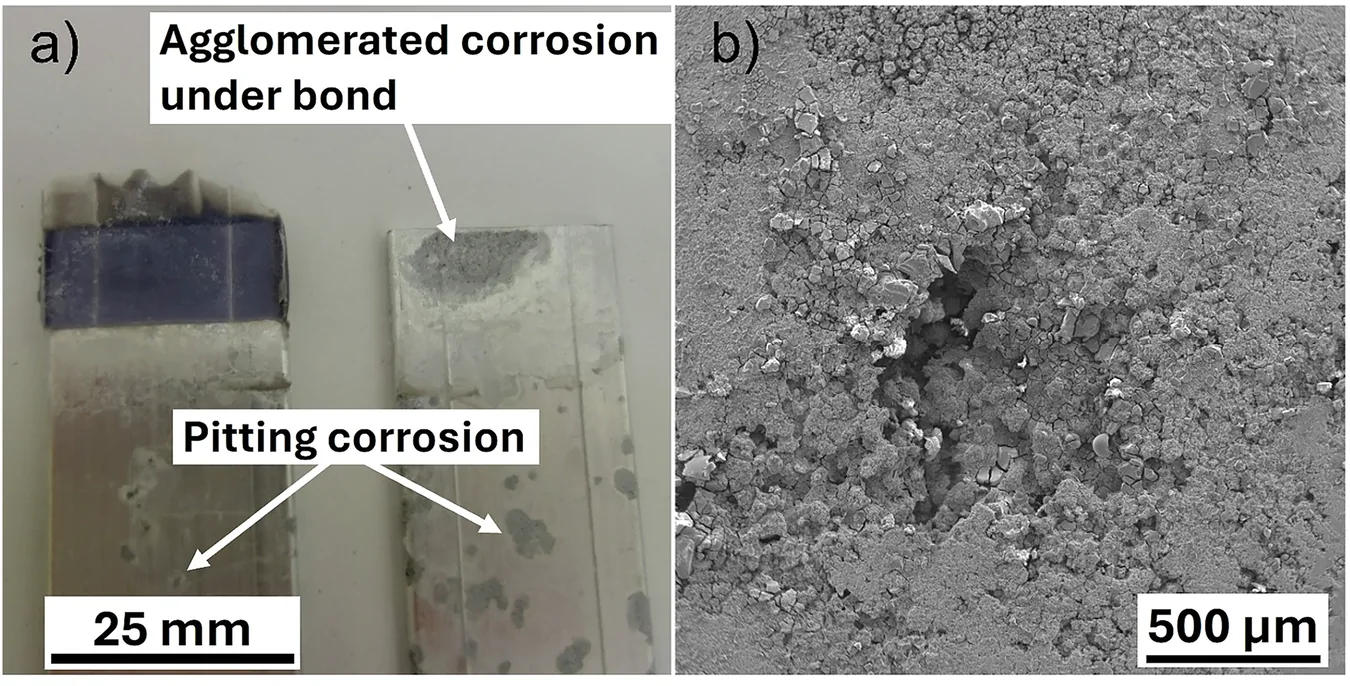
SWAAT test results for uncoated AA6060-T6. **a** Corrosion observed on spontaneously debonded AA6060-T6 coupons following 336 h SWAAT exposure. **b** SEM micrograph of pitting corrosion observed on the same surface.

It was apparent from testing that the most important attribute of the surface condition for the promotion of long-term bond durability of AA6060-T6 is the corrosion resistance. During SWAAT exposure, bare untreated samples were seen to degrade rapidly. Signs of corrosion were observed after one day of SWAAT exposure, and the condition of coupons deteriorated up to the 336 h inspection interval, at which point all of the untreated samples were observed to have spontaneously de-bonded and were removed from the chamber. Severe pitting corrosion was observed across all of the untreated coupons (Fig. 19). Examination of the bond area revealed severe bond-line corrosion. Whilst pitting corrosion on the exposed areas of the coupon occurred sporadically in isolated locations, within the bond area, a large, agglomerated area of corrosion was observed (Fig. 19a). This would appear to be caused by electrolyte entrapment beneath the adhesive, akin to crevice corrosion^76^. Corrosion at the epoxy/oxide interface can originate from diffusion transport of the electrolyte to the interface, where H_2_O can hydrogen bond at the interface at the expense of hydrogen bonding between adhesive and adherent^77^, or it can occur due to corrosion occurring at the edge of the joint sequentially passing into the bond area^78^. Based on the location of the agglomerated area adjacent to the edge of the bond and due to the speed at which deterioration of the bond progressed, it is suspected that primary corrosion commenced at the edge of the joint and propagated inwards. It is believed that aggressive changes to the Cl^−^ concentration and oxygen differentials in the localised electrolyte that seeped into the bond line, facilitated by the crevice-like conditions^79^ may have contributed to advancing the speed of catastrophic corrosive attack in the bonded area.

**Fig. 20:**
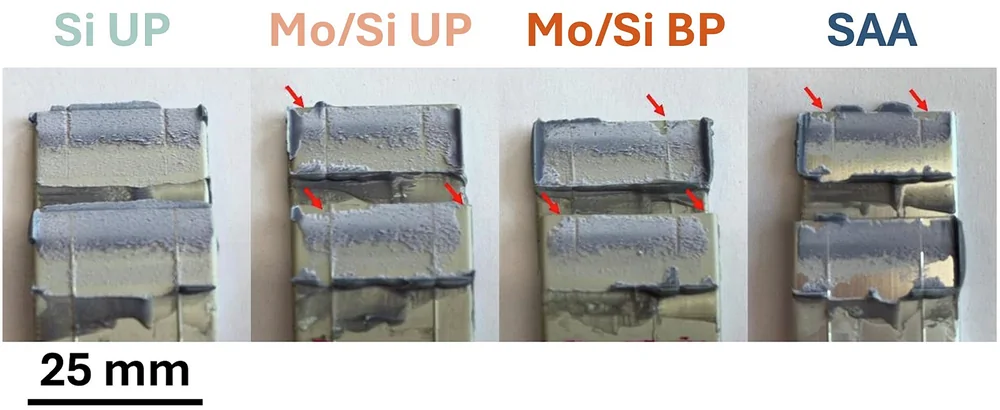
Fracture surfaces of adhesive joints after 1008 h SWAAT tests . Fracture surfaces obtained after 1008 h SWAAT tests of flash-PEO and SAA-treated AA6060-T6 samples. Red arrows show localised areas of interfacial failure.

No visible signs of corrosion were present on the coated areas on any of the PEO-treated or SAA-treated samples after 1008 h, indicating that the coatings were effective in providing protection from apparent pitting corrosion. The lap shear test results obtained for aged joints following SWAAT exposure are displayed in Fig. 16 along with the reduction in bond strength, quantified as a percentage. All treated samples exceeded the industry benchmark requirement of maintaining a minimum of 70% of the initial lap shear strength after 1008 h SWAAT exposure^6,71^. All PEO-treated lap shear joints exhibited a comparable or enhanced average strength compared to the sealed SAA-treated joints after ageing and matched or exceeded the latter in retained joint strength percentage. The Si UP-treated sample exhibited the highest lap shear strength of 31.5 ( ± 1.0) MPa, whilst UP and BP Mo/Si PEO-treated joints recorded average strengths of 28.9 (±0.9) and 26.8 (±0.8) MPa, respectively, and SAA-treated samples failed at an average strength of 28.5 (±0.4) MPa. The fracture surfaces obtained from PEO and SAA-treated samples (Fig. 20) indicate that samples generally failed in the optimal 50/50 close to surface mode; however, local defect sites where the fracture path deviates to the interface could be observed (labelled by red arrows), signifying points of interfacial weakness.

The modest reduction in bond strength observed across all treated samples could in part be attributed to degradation in the bulk properties of the epoxy adhesive. This can occur due to moisture uptake, which can act as a plasticiser, resulting in volumetric changes and hydrolysis that could both diminish the mechanical properties of the adhesive^80,81^. It is likely that a partial contribution to the reduction in strength over 1008 h SWAAT exposure is localised interfacial weakening and that this is likely the key differentiating factor, governing the modest differences in post ageing strength to failure in samples treated via different coating processes. The local interfacial defect sites (highlighted in Fig. 20) were less numerous on Si UP PEO-treated samples, which showed the lowest reduction in lap shear strength after ageing. As there were no apparent signs of primary corrosion, it is most likely that localised adhesive defect sites were caused by the presence of moisture at the adhesive/adherend interface. These local interfacial weaknesses appeared to be mostly located near the edges of the bond, to which moisture could diffuse most readily due to proximity to free surfaces of the epoxy. When present at the interface, moisture can disrupt interfacial bonds between epoxy and the adherend surface as well as hydrate the oxide surface^82^.

The two surface phenomena most closely associated with interfacial strength are the surface chemistry and the surface morphology. Whilst XPS revealed some variance in the surface chemistry, the topographies of the coatings varied significantly, and this is likely to be significant in determining the bond durability. The aged bond strength obtained for corresponding PEO treatments ranked in accordance with surface roughness (*R*_a_). One hypothesis to explain this is that the increased surface roughness of the Si UP PEO-treated samples associated with the presence of globular surface features afforded enhanced mechanical interlocking, resulting in superior bond durability. This is supported by previous research suggesting that the role of mechanical interlocking becomes more influential as adsorption weakens with ageing^83^. A counter hypothesis for the difference in the obtained bond strengths after ageing lies in the wettability of the surfaces produced via different PEO treatments, which also rank in accordance with the obtained strength after aging. Enhanced wettability of the surface may result in fewer interfacial voids, which could act as sites for moisture to collect at the interface, from which moisture attack of the interface components can occur. Whilst there is no conclusive evidence as to which of these hypotheses is responsible, it appears likely in either case that the topography of the flash-PEO oxide surface plays a significant role in the bond strength of adhesively bonded flash-PEO treated aluminium after exposure to a corrosive environment. It should also be highlighted that Si UP-treated samples exhibiting the highest bond durability in SWAAT exposure testing exhibited the lowest charge-transfer resistance in EIS measurements. This signifies that even flash-PEO coatings exhibiting the lowest electrochemical impedance displayed substantial corrosion resistance to negate the apparent influence of corrosion in demanding environmental conditions.

## Discussion

In summary, we produced uniform flash-PEO coatings of low thickness under different electrolyte/process conditions. The coatings, which exhibited differing surface morphologies, were extensively characterised and investigated with respect to adhesive bond durability, which resulted in the following findings:

- The addition of Na_2_MoO_4_ to the silicate electrolyte reduced the edge effect in the PEO process under high-frequency pulsed bipolar polarisation, enabling the uniform treatment of aluminium via bipolar treatments under atypically short process durations.
- Flash-PEO coatings featured differing surface morphologies. Mo/Si BP coatings were characterised by a lower density of globular features and a higher density of smaller open micropores, altogether leading to a reduced surface roughness. Mo/Si UP and Si UP coatings exhibited a greater density of globular features and higher surface roughness in increasing order.
- The Mo/Si BP flash-PEO-treated surfaces exhibited improved corrosion resistance, displaying higher kinetic charge-transfer resistance than other PEO coatings investigated and a large diffusional impedance component, both in line with the potential role of molybdenum in corrosion processes and the observed differences in coating morphology.
- As-new PEO-treated samples demonstrated lap shear strengths comparable to those of conventional sealed sulphuric acid-treated AA6060, and all fractured in a preferable failure mode, within the adhesive phase. Mo/Si BP-treated samples demonstrated the highest lap shear strength of the flash-PEO treatments investigated.
- All PEO coatings demonstrated satisfactory corrosion resistance in SWAAT testing and exhibited comparable or better aged lap shear durability after ageing, than sealed sulphuric acid anodised AA6060-T6.
- Whilst all PEO treatments demonstrated satisfactory bond durability, it was identified that the Si UP PEO-treated samples exhibited the lowest reduction in bond strength after ageing, which could be attributed to an increase in mechanical interlocking or wettability, both of which are resultant from an increased surface roughness.

The results obtained from the investigation into flash-PEO coatings demonstrated that such surface treatments can provide higher initial bond strength than dense, thicker conventional PEO coatings assessed in previous studies and can provide comparable or superior bond durability compared to industrial standard sealed SAA treatments when subjected to accelerated ageing conditions. Based on performance, flash-PEO technology has a promising outlook as a potential alternative technology to conventional sulphuric acid anodising.

## Methods

### Materials

Surface treatments were carried out on the alloy AA6060-T6 (wt% composition: Si 0.51; Mg 0.4; Fe 0.21; Mn 0.05; Cu 0.03; Ti 0.02; Ni 0.01; Al balance), an alloy typically utilised for structural automotive components. Characterisation studies were carried out on samples of dimensions 20 mm × 25 mm × 2 mm (surface area = 11.8 cm^2^) ground up to P1200 grit finish. Lap-shear testing was performed on extruded AA6060-T6 samples of 100 mm × 25 mm × 2 mm with extruded 0.2 mm spacers to ensure uniform bond thickness across testing. The extruded samples were masked so that only 11.8 cm^2^ area was exposed at the end to minimise current and potential distribution variations that geometrical changes could impose on the process.

### Flash-PEO process

Flash-PEO surface treatments were carried out as supplied substrates degreased with alcohol, in two electrolyte baths referred to as Si and Mo/Si hereafter. The former consisted of 0.05 M KOH and 0.118 M Na_2_SiO_3_ for a conductivity of 15.6 mS cm^-1^ and pH 12.44. The latter electrolyte additionally contained 0.025 M Na_2_MoO_4_ for a conductivity of 19.1 mS cm^-1^ and pH 12.42. The treatments were carried out in a 4-litre glass beaker equipped with a stainless-steel collar and helical water-cooling channel that acted as the counter electrode. The electrolyte temperature was maintained in the range of 10–20 °C. The polarisation was provided by a Magpuls 1000/35/200BP power supply (Magpuls GmbH, Germany) equipped with 2 ADL GX 100/1000 DC power supply units (ADL GmbH, Germany). Transient voltage and current signals were acquired using current and voltage probes fed back to a NI PXI-5922 oscilloscope, monitored, and recorded in an NI LabVIEW environment. The treatments were carried out under pulsed unipolar (UP) and bipolar (BP) conditions at 2.5 kHz. Each pulse duration was 150 μs followed by a pause of either 250 (UP) or 50 μs (BP). The current density was set at 68 mA cm^−2^ in both anodic and cathodic pulses. The treatment process included a 10 s potentiodynamic ramp period to protect the power supply before switching to a 60 s hold at the designated anodic and cathodic current densities.

Cyclic voltammetry studies were carried out using the same cell configuration with an MDX II DC power supply (Advanced Energy Industries, Inc.). Additional experimentation was carried out using varying concentrations of sodium silicate to demonstrate the effect of electrolyte chemistry on PEO phenomenology.

### Sealed SAA coatings

Type II sulphuric acid anodising treatments were carried out in a commercial anodising line at BCW Treatments Ltd. AA6060-T6 coupons were degreased, alkaline etched and desmutted prior to anodising in a 15 wt% aqueous solution of sulphuric acid at 18–22 °C. The anodising was carried out at a constant current density of 140 A m^-^^2^ (voltage range of 20–25 V). The SAA samples were subsequently sealed via a hydrothermal post-treatment for 20 min at 96 °C.

### Materials characterisation

Topological and cross-sectional morphologies were observed using a Tescan Mira 3 scanning electron microscope equipped with an Oxford Instruments energy dispersive X-ray (EDX) detector for elemental analysis. Coating cross-sections were prepared on samples mounted in epoxy resin by progressive grinding with 240–3000 grit emery paper, followed by polishing up to 0.25 μm diamond paste finish. The imaging was performed under 10 kV acceleration voltage in back scattered and secondary electron modes for cross-sectional and surface plane views, respectively. Coating thicknesses were measured via SEM projection of the cross-section. EDX analysis was carried out using Aztec software (Oxford Instruments). The surface roughness was evaluated using a Dektak XT stylus profilometer (Bruker), taking triplicate measurements over 1000 μm length. The contact angle was measured with a DSA100 drop shape analyser (Kruss) using one-microlitre deionised water droplets, with results presented as the mean average ± standard deviation of minimum three measurements.

Phase composition analysis of the investigated coatings was performed using grazing-incidence angle X-ray diffraction (GA-XRD). Measurements were performed at a grazing angle of *α* = 1° for flash-PEO and *α* = 2° for SAA using a Bruker D8 Advance Diffractometer (Cu K-alpha radiation). XRD patterns were analysed using Panalytical X’pert Highscore software.

Surface chemical composition was analysed using X-ray photoelectron spectroscopy (XPS). XPS was performed using an axis ultra hybridge spectrometer (Kratos Analytical) using monochromated Al Kα radiation (1486.6 eV, 10 mA emission at 150 W and a spot size of 300 × 700 µm) and a vacuum pressure of 5 × 10^−9^ mbar. The pass energy for survey and high-resolution scans was set at 160 and 20 eV, respectively. Data calibration was carried out using the carbon 1*s* peak at 285 eV. Spectra were analysed and fitted using CasaXPS software.

### Electrochemical evaluation

Electrochemical studies of coated substrates in this work were auxiliary to the long-term durability tests of the bonded structures. The studies were conducted in a naturally aerated 3.5% NaCl aqueous solution in ambient conditions using a VSP-300 potentiostat (Biologic, France). The studies were performed using a three-electrode cell, 200 mL in volume, including a platinum counter electrode, an Ag/AgCl reference electrode (3.5 M KCl, +0.205 V vs. SHE). The studied sample, which served as the working electrode, was pressed upwards against a circular orifice at the bottom of the cell, ensuring an exposed surface area of 1 cm^2^. The OCP was measured after 1, 3, 24, 48 and 72 h of sample exposure to a corrosive environment. Since the targeted applications do not imply permanent immersion in corrosive media, this was deemed sufficient to evaluate mechanisms of coating degradation and their contribution to the joint durability. Electrochemical impedance spectroscopy was conducted at OCP using a 10 mV sinusoidal perturbation at a frequency range of 92 kHz to 10 mHz. The spectra were recorded at 3, 24 and 72 h to assess the change in impedance response over time. Equivalent circuit fitting of the impedance data was performed using Zview software (Scribner Associates Inc.).

### Lap shear testing and durability testing

Lap shear tests were carried out in accordance with ASTM D1002^84^. Coated coupons for lap shear testing were bonded together with the heat-cured epoxy, Betamate 4600F (DuPont de Nemours, Inc.), using a bond area of 10 mm × 25 mm and a bond thickness of 0.2 mm. Bonded coupons were cured via a multistage heating procedure (see Supplementary Materials, Fig. S6). To assess the bond strength, the bonded coupons were loaded in the tensile direction at an extension rate of 10 mm min^-1^, using an Instron 3367 load frame. Comparative untreated AA6060-T6 coupons were prepared via cleaning with solvent (isopropanol) and then deionised water.

To analyse the long-term durability, lap shear joints were subjected to an accelerated environment, Sea Water Acetic Acid Test (SWAAT) for 1008 h according to ASTM G85 (Annex A3)^85^. This was performed using a C&W SF/MP450 salt spray chamber. The test consisted of repeated 2-h cycles comprising a 30 min spray followed by a 90 min soak period at 98% humidity. The SWAAT solution was composed of an artificial seawater with the addition of 10 mL L^-1^ glacial acetic acid and pH adjusted to the range of 2.8–3 using NaOH. The cabinet temperature was maintained at 49 °C. To assess the change in bond strength following exposure to the corrosive environment, the surviving bonded samples were subjected to the lap shear tests under identical conditions.

## Supplementary information

Supplementary materials

## Data Availability

All data supporting this study are provided in sections ‘Results’ and ‘Discussion’ and ‘Supplementary Materials’.
