# Supplementary material for: Adhesive bond durability and corrosion resistance of AA6060-T6 flash-PEO treated in Mo-Si solutions
Source: Npj Mater Degrad. 2026 Jul 15;10(1):134. doi: 10.1038/s41529-026-00841-5 (PMC13739740; doi:10.1038/s41529-026-00841-5)
Supplement: Supplementary file 1 [file 41529_2026_841_MOESM1_ESM.pdf]

Supplementary Materials for

Adhesive Bond Durability and Corrosion Resistance of AA6060-T6 Flash-PEO Treated in Mo-Si Solutions

Dominic Shore [1,2], *Junia C. Avelar-Batista Wilson* [3], *Nicolas Laugel* [1,2], *Allan Matthews* [1,2], *Aleksey Yerokhin* [1,2,*]

[1] Department of Materials, University of Manchester, Oxford Road, Manchester, M13 9PL, UK

[2] Henry Royce Institute, University of Manchester, Manchester M12 9PL UK

[3] BCW Treatments Ltd, 3C Innovation Drive, Bancroft Road, Burnley, Lancashire BB10 2FT, UK

* Corresponding author: Aleksey.Yerokhin@manchester.ac.uk (A. Yerokhin

Voltage transients of flash-PEO process

Voltage-time plots for the unipolar and bipolar PEO process treatments in Si and Mo/Si electrolytes are displayed in **Figure S1**. Comparing the anodic voltage response, the transient voltage is higher in the case of the Si electrolyte for both the unipolar and bipolar processes. The decrease in the anodic cell voltage in Mo/Si electrolyte may be attributed to its higher conductivity contributing to a decreased Ohmic drop across the electrolyte. Comparing the anodic voltage during the unipolar and bipolar treatments, it is observed to be lower in the latter process for both electrolytes. There can be multiple explanations for this observation. Firstly, oxide growth rate may be marginally lower in the bipolar process compared to its unipolar counterpart, leading to a slightly lower electrical resistance of corresponding oxide layer and secondly, differential coating porosity with differential process variables may be influential. This could also be attributed to the effect of barrier layer hydration induced by cathodic polarization[1] on the coating resistance to anodic polarisation.

As is accustomed with the formation of oxides on valve metals, the cathodic voltages in the bipolar processes are significantly lower than corresponding anodic voltages due to the asymmetric current response of the metal-oxide electrolyte junction (see reference 13 in the main text). It is observed that in the latter part of the treatment, the cathodic voltage raises to higher amplitude in the Mo/Si electrolyte than the Si electrolyte, despite the higher ionic conductivity of the Mo/Si electrolyte, this could be the result of more unform coating coverage providing a more substantive barrier to current.

1. Rogov, A. B., Matthews, A. & Yerokhin, A. Role of cathodic current in plasma electrolytic oxidation of Al: A quantitative approach to in-situ evaluation of cathodically induced effects. Electrochimica Acta 317, 221–231 (2019).

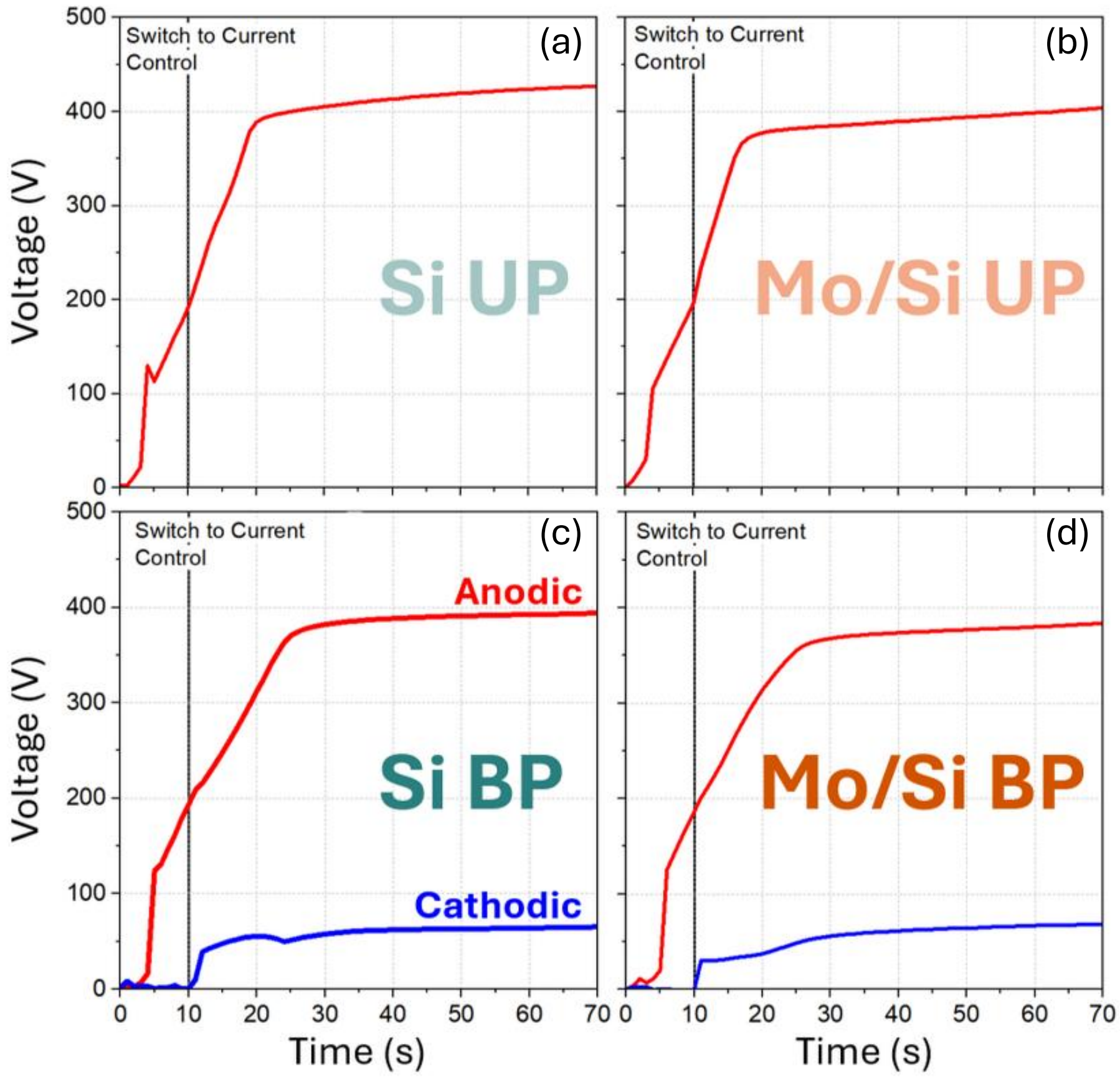

Figure S1: Voltage time evolution for investigated set of flash-PEO conditions. (a) Si UP, (b) Mo/Si UP, (c) Si BP, (d) Mo/Si BP.

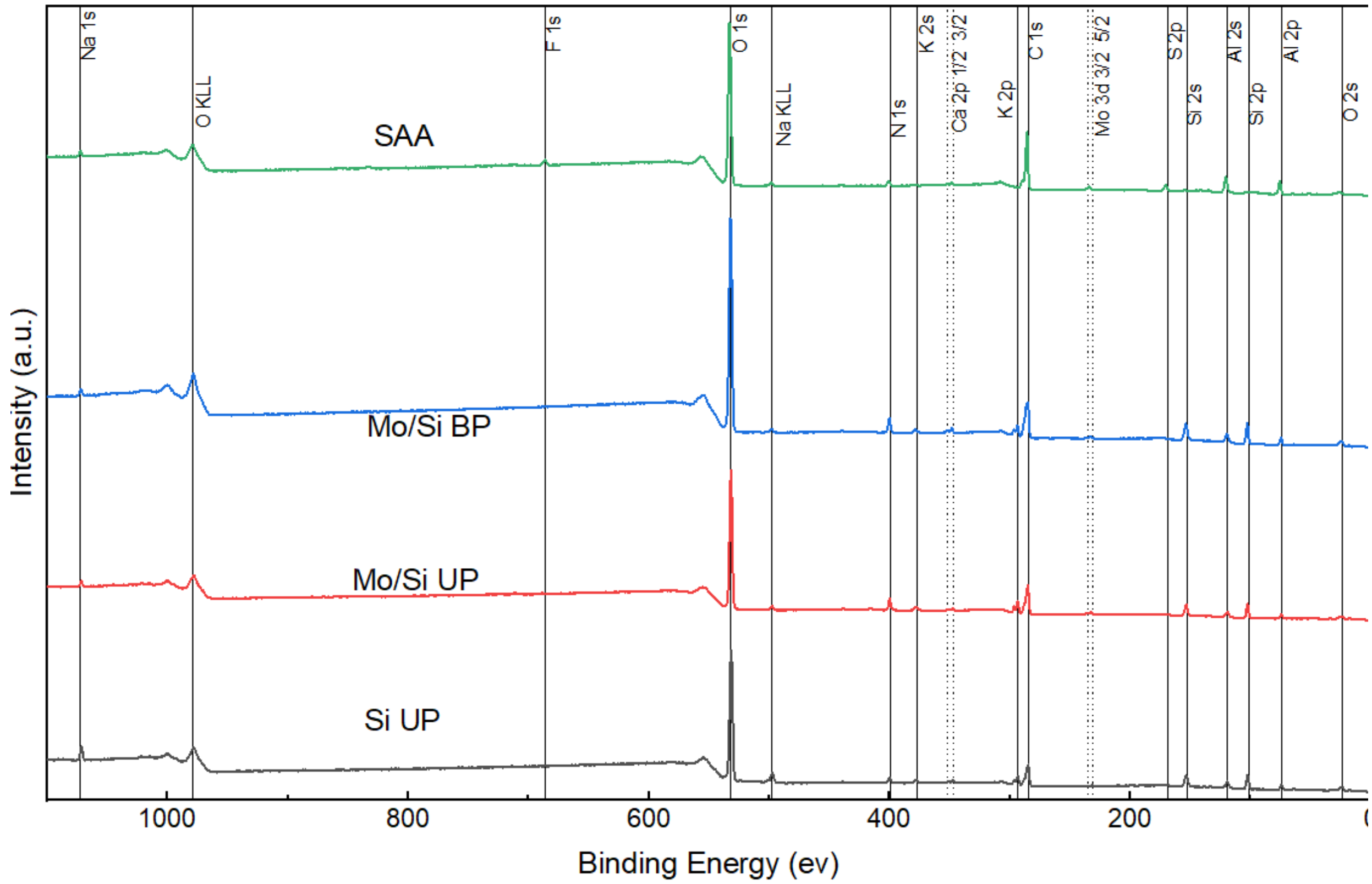

Figure S2: **Survey scans of samples** treated by Si UP, Mo/Si UP, Mo/Si BP flash-PEO and SAA.

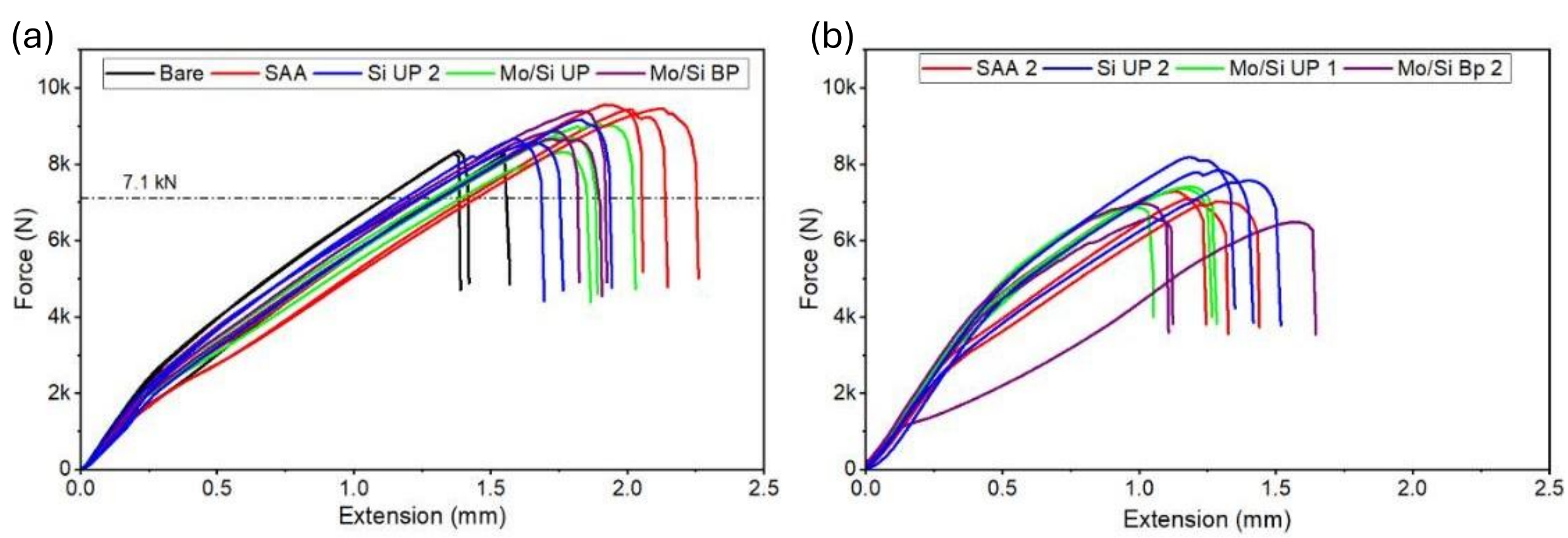

Figure S3: **Force/extension curves for the studied adhesively bonded samples** in un-weathered #conditions (a) and after 1008h SWAAT exposure (b).

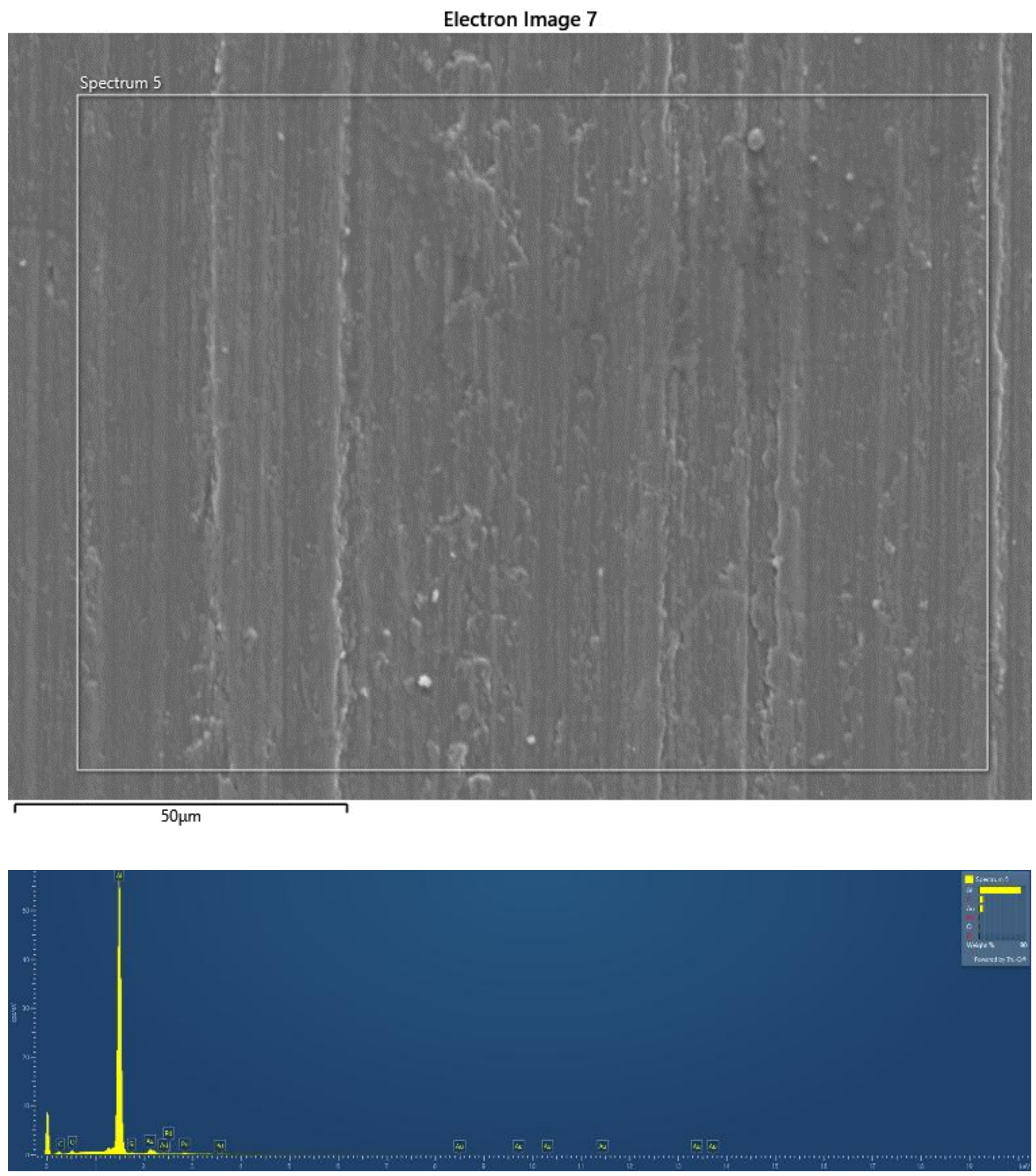

Figure S4: EDX spectrum and measurement sample area of aluminium region showing clean interfacial failure.

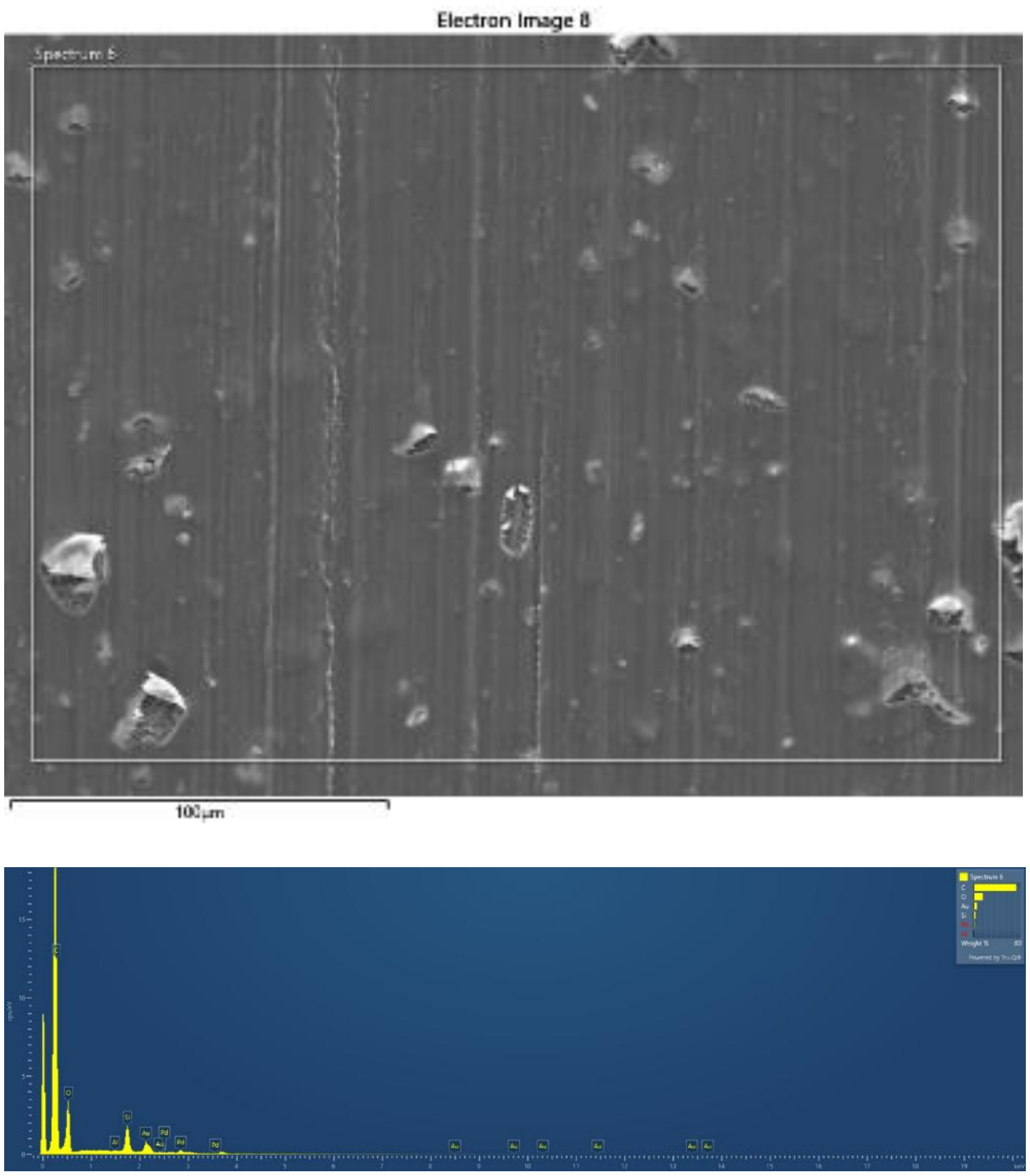

Figure S5: EDX spectrum and measurement sample area of epoxy fracture surface.

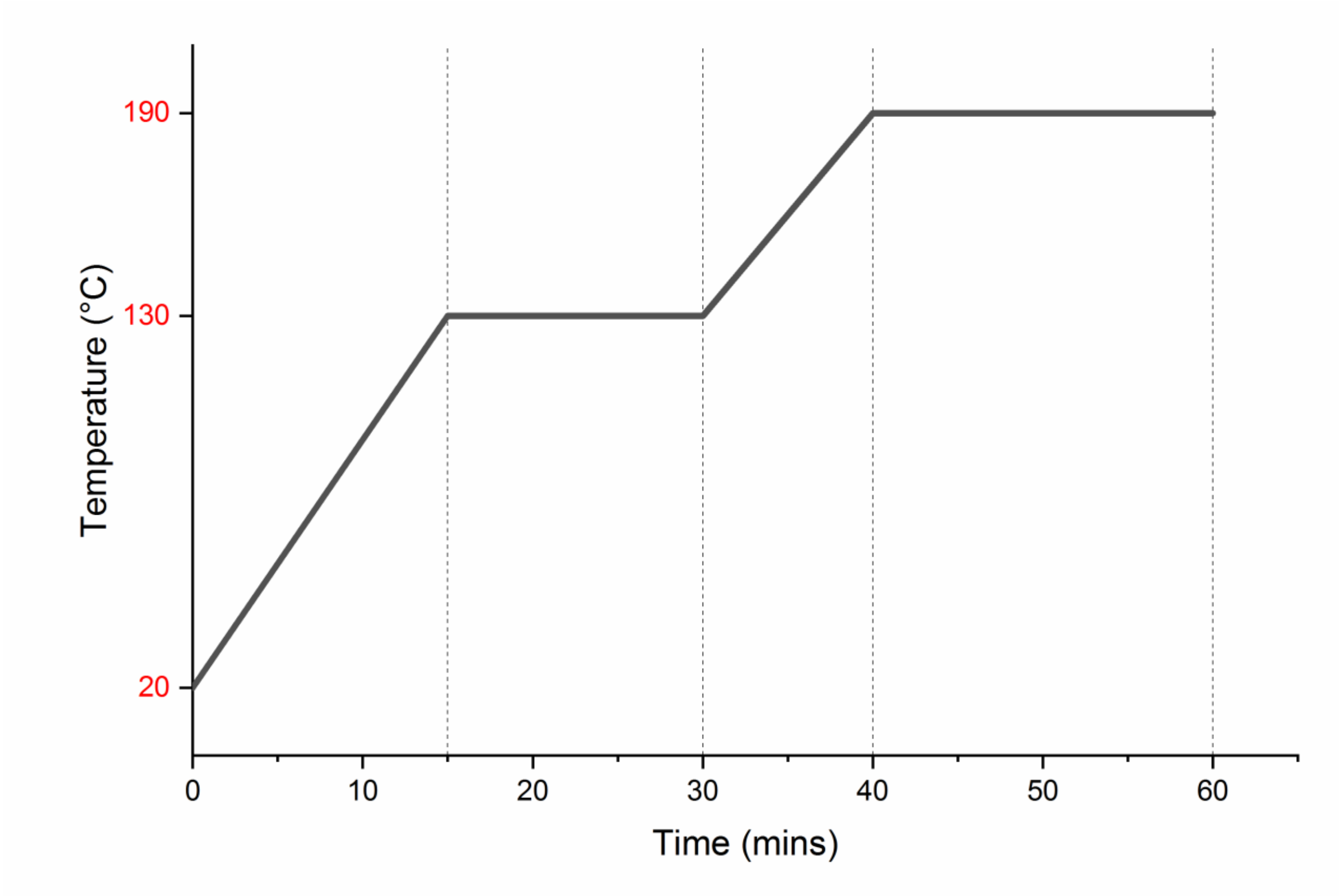

Figure S6: Heat profile for curing of Dow 4600F adhesive.

Table S1: EIS parameters used to fit data in Figure 12 and Figure 13. Equivalent circuits are presented in Figure 14 of the main article. Bare, 3h: R3 = 6.41 kΩ cm^2; L3 = 5.24 kΩ s cm^2.

		CPE1	CPEn1	R1	CPE2	CPEn2	R2	Ws-R	Ws-T	Ws P	$R_{p\text{-total}}$
		$\Omega^{-1}s^{n}cm^{-2}$		Ωcm^2	$\Omega^{-1}s^{n}cm^{-2}$		Ωcm^2	Ωcm^2	s		Ωcm^2
**Bare**	3 h	6.75 µ	0.858	1.44 k	1.75 µ	1.000	14.0 k				4.52 k
	24 h				14.0 µ	0.825	15.6 k	12.1 k	139	0.612	15.6 k
	72 h				12.7 µ	0.847	35.7 k	6.76 k	45.2	0.609	35.7 k
**Si UP**	3 h	86.2 n	0.833	71.4 k	0.86 µ	0.792	391 k				463 k
	24 h	140 n	0.809	94.1 k	1.06 µ	0.807	234 k				328 k
	72 h	161 n	0.809	69.6 k	1.53 µ	0.732	326 k				395 k
**Mo/Si UP**	3 h	10.8 n	1.000	1.80 k	54.8 n	0.739	4.14 M	31.0 M	1,110	0.500	4.14 M
	24 h	10.4 n	0.988	0.53 k	36.0 n	0.900	646 k	70.9M	2,070	0.428	647 k
	72 h	43.4 n	0.885	1.05 k	11.6 n	0.988	1.17 M	81.9 M	1,020	0.478	1.17 M
**Mo/Si BP**	3 h	42.9 n	0.914	131 k	38.4 n	0.721	7.33 M	46.5 M	1,930	0.495	7.46 M
	24 h	67.0 n	0.871	484 k	4.72 n	1.000	1.96 M	52.9 M	1,200	0.322	2.44 M
	72 h	613 n	0.879	300 k	6.17 n	1.000	13.7M	144 M	102	0.475	14.0 M
		**CPE1**	**CPEn1**	**N/A**	**CPE2**	**CPEn2**	**R2**	**CPE3**	**CPEn3**	**N/A**	**$R_{p\text{-total}}$**
		$\Omega^{-1}s^{n}cm^{-2}$			$\Omega^{-1}s^{n}cm^{-2}$		Ωcm^2	$\Omega^{-1}s^{n}cm^{-2}$			Ωcm^2
**SAA**	3 h	2.13 n	1 (c)		167 n	0.534	143 k	534 n	0.934	-	*
	24 h	1.92 n	1 (c)		201 n	0.532	138 k	535 n	0.926	-	*
	72 h	1.66 n	1 (c)		208 n	0.534	135 k	525 n	0.918	-	*
